# Aggregation-Induced Emission with Alkynylcoumarin Dinuclear Gold(I) Complexes: Photophysical, Dynamic Light Scattering, and Time-Dependent Density Functional Theory Studies

**DOI:** 10.1021/acs.inorgchem.2c00366

**Published:** 2022-04-27

**Authors:** Carla Cunha, Andrea Pinto, Adelino Galvão, Laura Rodríguez, J. Sérgio Seixas de Melo

**Affiliations:** † CQC-IMS, Department of Chemistry, University of Coimbra, Rua Larga, Coimbra 3004-535, Portugal; ‡ Departament de Química Inorgànica i Orgànica, Secció de Química Inorgànica, Universitat de Barcelona, Martí i Franquès 1−11, Barcelona E-08028, Spain; § Institut de Nanociència i Nanotecnologia. Universitat de Barcelona, Barcelona 08028, Spain; ∥ Centro de Química Estrutural, Institute of Molecular Sciences, Instituto Superior Técnico, Universidade de Lisboa, Av. Rovisco Pais, 1049 001 Lisboa, Portugal

## Abstract

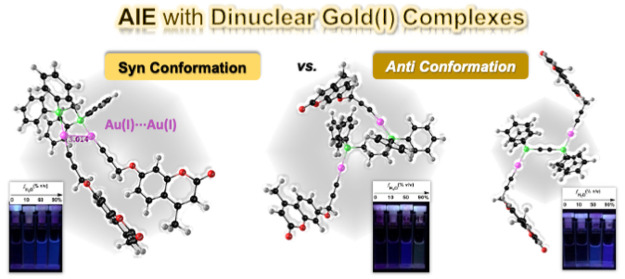

Aggregation-induced emission (AIE) has gained a remarkable amount of interest in the past 20 years, but the majority of the studies are based on organic structures. Herein, three dinuclear gold(I) complexes, with the general formula [PPh_2_XPPh_2_-Au_2_-Coum_2_], where the Au(I) atom is linked to three different diphosphanes [PPh_2_XPPh_2_; DPPM for X = CH_2_ (**1.1**), DPPP for X = (CH_2_)_3_ (**1.2**), and DPPA for X = C≡C (**1.3**)] and the propynyloxycoumarin precursor (**1**, 4-methyl-substituted coumarin), have been synthesized. The compounds present AIE characteristics, AIEgens, with high luminescence quantum yields in the solid state when they are compared to dilute solutions. Photophysical studies (steady-state and time-resolved fluorescence) were obtained, with AIE being observed with the three gold(I) complexes in acetonitrile/water mixtures. This was further corroborated with dynamic light scattering measurements. Time-dependent density functional theory (TDDFT) electronic calculations show that the compounds have different syn and anti conformations (relative to the coumarin core) with **1.1** syn and **1.2** and **1.3** both anti. From time-resolved fluorescence experiments, the augment in the contribution of the longer decay component is found to be associated with the emission of the aggregate (AIE effect) and its nature (involving a dimer) rationalized from TDDFT electronic calculations.

## Abstract 2

Aggregation-induced emission (AIE) found with three dinuclear gold(I) complexes [PPh_2_XPPh_2_-Au_2_-Coum_2_], where X = DPPM (**1.1**), DPPP (**1.2**), and DPPA (**1.3**). This was obtained from steady-state and time-resolved fluorescence experiments in acetonitrile/water mixtures, further complemented by dynamic light scattering experiments. Different syn and anti conformations are found and the double-exponential decay associated with the emission of the aggregate (AIE effect) and its nature (involving a dimer) rationalized from time-dependent density functional theory electronic calculations.

## Introduction

Since the pioneering study of Tang and co-workers, there has been a substantial increase in the number of luminescent compounds with aggregation-induced emission (AIE) properties (AIEgens).^1−7^ One current strategy to improve the luminescence emission in the solid state involves the development of structures, whereby when molecular movements, free motion, or internal rotation (loose bolt or rigid rotor effects) of a molecule are restricted, the radiative deactivation increases because of a decrease of the radiatiotionless deactivation channel.^7^ This is valid for excited singlet and triplet states.^8−10^ AIEgens, such as tetraphenylethylene (TPE), show weak emissions in dilute solutions (good solvents) but emit intensely when they aggregate and, usually, also in the solid state (aggregates). The AIE effect has been linked to the restriction in intramolecular rotations, by aggregate formation, of the emissive molecules, which efficiently limits the radiationless energy deactivation, enhancing the radiative decay.^7,11^ With AIEgens, the particular shape of the TPE units prevents luminogens from packing (in the luminescence loss π–π stacking), while the intramolecular rotations (an efficient route for radiationless deactivation through the loose bolt effect) are physically constrained in the condensed phase. Aggregates typically have dimensions around 100 nm, and its luminescence efficiency is also found to depend on the degree of crystallinity.

A growing interest in organometallic complexes has been observed in the past 2 decades mainly because of their potential use in a variety of applications; in particular, gold(I)-derived complexes represent an area of research that is emerging mainly because of their luminescent properties, both in the solid state and in solution.^12−15^ Gold(I) complexes are particularly interesting because of the structural characteristics of their ligands but also because of the possible establishment of Au^I^···Au^I^ interactions (an aurophilicity phenomenon),^12,16−18^ which can modulate and give the resulting assembly properties and various potential applications in, for example, drugs, photodynamic therapy (PDT) agents, and sensors.^19−23^ This interaction (aurophilicity) can be of intramolecular or intermolecular origin, and the ideal distance for such an interaction to be significant must be lower than, or close to, the sum of the van der Waals radii (3.32 Å),^24,25^ and the energy of these bonds is analogous to that of strong hydrogen bonds (5–10 kcal/mol).^24,26^ This synergy can provide additional stability to the complexes derived from gold(I).^27^ Self-assembly in supramolecular aggregates can facilitate other types of interactions between ligands, including π–π-stacking interactions, that can also involve “neighboring” complexes, which, in turn, can lead to new emission bands, as a result of AIE.^6,28−31^ In this context, this class of compounds is one of the most promising in the current panorama of materials science.^24,26^ The luminescent properties of alkynylgold(I) complexes have grown significantly in the past several years.^27,32^ The general strategy for obtaining luminescent alkynylgold(I) complexes relies on the fact that the ligand is actually a luminophore of its own, whose emission in the triplet state increases strongly as a consequence of the heavy effect induced by gold.^27,29^ This, in turn, facilitates access to excited triplet states by increasing the spin–orbit coupling (SOC), thus favoring the S_1_∼∼→T_1_ intersystem crossing pathway.^8,23,30,32^

Inclusion of the phosphane ligands allows the introduction of complementary electronic properties, together with several different types of coordination geometries, in transition-metal centers, improving the predictable physical and chemical properties of these new complexes.^33−35^ Additionally, gold(I) complexes can display dual luminescence at room temperature with emission efficiencies that depend significantly on the structure of the molecule.^31,34,36^

**Scheme 1:**
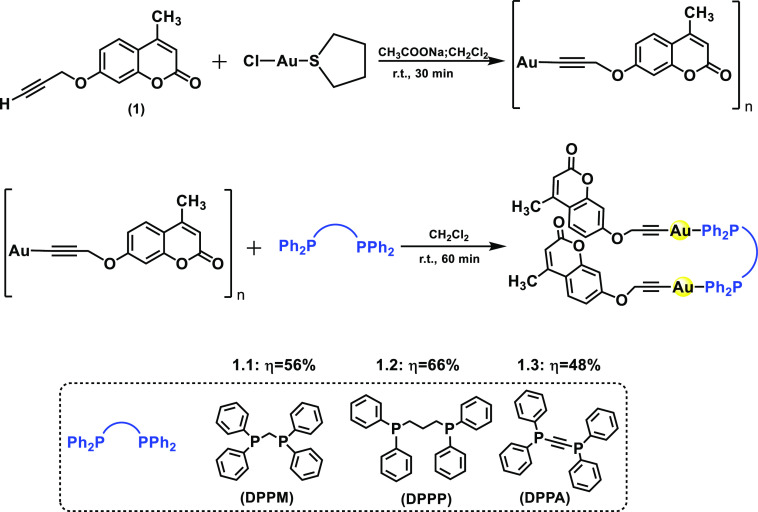
General Synthetic Routes, Structures, and Acronyms of Alkynylcoumarin Dinuclear Gold(I) Complexes [Au (C≡C_13_H_9_O_3_)(PPh_2_XPPh_2_)]_2_ [PPh_2_XPPh_2_ = DPPM for X = (CH_2_) (**1.1**), DPPP for X = (CH_2_)_3_ (**1.2**), and DPPA for X = C≡C (**1.3**)]

Despite some recent publications reporting gold(I) AIEgen complexes, with relevant works pointing to a diverse number of applications,^31,37−41^ including the potential to reach an efficient low dose of X-ray-induced PDT,^42^ only a very limited number of these offer a rationale of the observed phenomena with different techniques. In previous works of our group, we have investigated the alkynylcoumarin ligand, which presents different electron-donating and electron-withdrawing substituents and the water-soluble phosphane spacers 1,3,5-triaza-7-phosphaadamantane (PTA) and 3,7-diacetyl-1,3,7-triaza-5-phosphabicyclo[3.3.1]nonane (DAPTA).^32^ The 7-substituted coumarin chromophore (with the substitution of O–R, with R = alkyl group, in the 7 position) displays high fluorescence emission, making it highly appropriate to be investigated with fluorescence techniques.^32^ In this work, we describe the synthesis and analytical and photophysical (including steady-state and time-resolved data) characterization of a series of dinuclear gold(I) complexes constituted by an alkynyl-4-methylcoumarin ligand and diphosphanes with various lengths and flexibilities (**1.1**, **1.2**, and **1.3** in Scheme 1). In addition, dynamic light scattering (DLS) and time-dependent density functional theory (TDDFT) electronic calculations were performed, aiming to shed light on the nature of the aggregates.

## Results and Discussion

### Synthesis and Structural Characterization

Three different organometallic dinuclear gold(I) complexes containing the diphosphanes DPPM (**1.1**), DPPP (**1.2**), and DPPA (**1.3**) were synthesized. This required the previous synthesis of the polymer [Au(C≡C_13_H_9_O_3_)]_*n*_ (**1a**) from deprotonation of the propargyloxycoumarin ligand and a subsequent reaction with AuCl(tht), as the gold(I) source (Scheme 1). Correct formation of the compound was evidenced by the disappearance of the terminal alkynyl proton of the organic chromophore (∼3303 cm^–1^) and the presence of the C≡C vibration (∼2935 cm^–1^) in the IR spectrum (Figures S1 and S2). Then, a dichloromethane suspension of **1a** was stirred with the different phosphanes (PPh_2_XPPh_2_) in a 2:1 ratio (Scheme 1), and the reaction was kept under stirring for 60 min at room temperature. Coordination of the corresponding diphosphanes (DPPM, DPPP, and DPPA) gave pale-yellow (**1.1**) and white (**1.2** and **1.3**) solutions that yielded the corresponding complexes in moderate yields after concentration and precipitation with *n*-hexane (∼50–70%).

Characterization of **1.1**, **1.2**, and **1.3** was obtained from different spectroscopic techniques (IR and ^1^H and ^31^P NMR spectroscopy and positive-ion electrospray ionization mass spectrometry [ESI-MS(+)], which concluded with the successful synthesis of the complexes. The corresponding ^1^H NMR spectra show the characteristic protons of the organic ligand, together with the characteristic protons of the different diphosphanes (Figures S3–S5). Furthermore, the methylene protons, close to the alkynyl group, are detected as a singlet instead of the doublet recorded in the organic ligand due to coordination to the metal atom. ^31^P NMR obtained in CDCl_3_ typically shows signals at 31.0, 34.3, and 17.0 ppm, which are ca. 50 ppm downfield-shifted compared with the free diphosphane, as expected for coordination to the gold(I) atom (Figures S6–S8). From IR spectroscopy, the characteristic *v*(C≡C) vibration was also observed at 2130 cm^–1^. Formation of the gold(I) complexes was finally confirmed by ESI-MS(+) measurements, with the detection of the corresponding molecular peaks [M + Na]^+^ and [M + K]^+^ of the protonated species for all complexes (Figures S9–S11).

### Electronic Spectral Characterization

**Figure 1:**
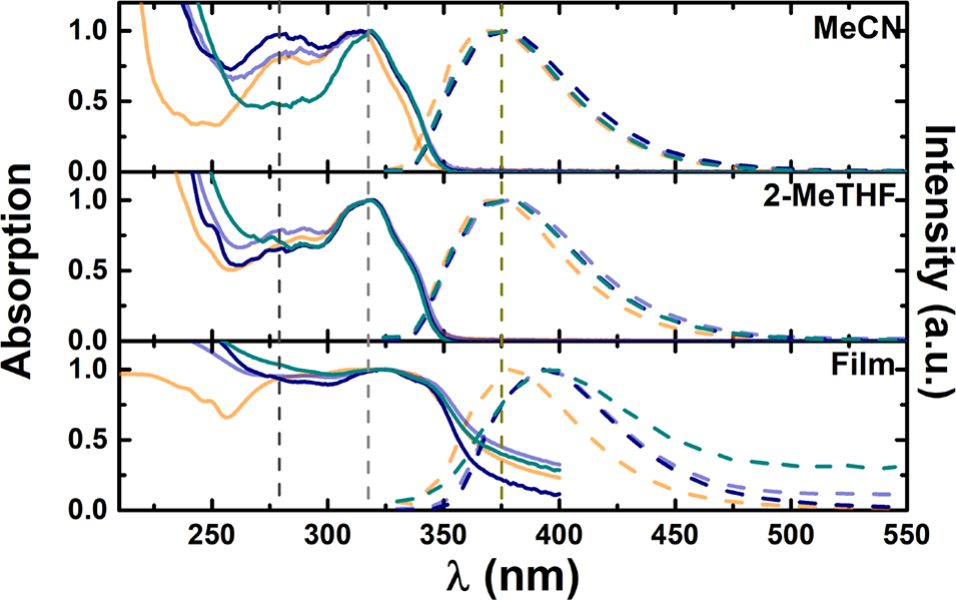
Absorption and fluorescence emission spectra of the dinuclear gold(I) complexes (**1.1**, **1.2**, and **1.3**) with the parent compound (**1**) in MeCN, 2-MeTHF, and thin films. Vertical dashed lines are just guides to the eye. Color legend for lines: absorption and emission spectra of **1**, orange; **1.1**, purple; **1.2**, blue; **1.3**, dark green.

The three dinuclear gold(I) complexes **1.1**, **1.2**, and **1.3** and the propynyloxycoumarin ligand **1** were further investigated, aiming to clarify the effect of the diphosphanes and the presence of gold(I) atoms on the photophysical properties and the presence of AIE. Figure 1 represents both the absorption and fluorescence emission spectra of **1.1**, **1.2**, and **1.3**, together with the corresponding data of **1** in different organic solvents and in the solid state (thin films) at room temperature (293 K). The relevant electronic spectral parameters (including the absorption, fluorescence, and phosphorescence emission wavelength maxima, fluorescence quantum yields, and Stokes shifts) are summarized in Table 1. The data for **1**, which was previously studied, are included for comparison.^32^

**Table 1:** Spectroscopic Data (Including Wavelength Absorption λ_abs_, Fluorescence λ_em_, and Phosphorescence Emission Maxima λ^Ph^), Together with the Fluorescence Quantum Yields ϕ_F_ (in the Presence of Molecular Oxygen, O_2_, and Saturated Nitrogen, N_2 sat._) and Stokes Shift Δ_SS_ (cm^–1^), for the Gold(I) Complexes **1.1**, **1.2**, and **1.3** and Ligand **1** in Different Organic Solvents (Dioxane, Dx, 2-Methyltetrahydrofuran, 2-MeTHF, Dimethylformamide, DMF, and Acetonitrile, MeCN) and in the Solid State (Thin Films)

			293 K					77 K
	solvent	ε′^a^	λ_abs_ (nm)	λ_em_ (nm)	Δ_SS_ (cm^–1^)	ϕ_F_(O_2_)	ϕ_F_(N_2 sat._)	λ^Ph^ (nm)
**1**	Dx	2.25	317	376	4950	0.012	0.015	
	2-MeTHF	7.58	318	371	4492	0.010	0.014	489^a^
	DMF	36.7	316	374	4908	0.021	0.027	
	MeCN^a^	37.5	316	374	4908	0.013	0.016	
	film		321	380	4837	0.181		
**1.1**	Dx	2.25	313	375	5282	0.011	0.014	
	2-MeTHF	7.58	320	380	4934	0.012	0.015	490
	DMF	36.7	319	377	4823	0.023	0.025	
	MeCN	37.5	318	373	4681	0.010	0.013	
	film		325	395	5453	0.054		
**1.2**	Dx	2.25	320	374	4512	0.018	0.023	
	2-MeTHF	7.58	318	377	4921	0.015	0.022	489
	DMF	36.7	320	376	4654	0.018	0.023	
	MeCN	37.5	314	376	5251	0.011	0.013	
	film		325	393	5324	0.122		
**1.3**	Dx	2.25	321	374	4415	0.023	0.029	
	2-MeTHF	7.58	318	378	4992	0.013	0.015	488
	DMF	36.7	321	375	4486	0.025	0.032	
	MeCN	37.5	319	374	4610	0.012	0.013	
	film		325	392	5259	0.113		

a For **1** in MeCN and 2-MeTHF, the data are from ref (32).

In organic solvents, the gold(I) complexes display a strong absorption band at ∼320 nm, attributed to the coumarin chromophoric unit^32^ and also indicative of a minor influence of the two gold atoms, and of the diphosphane units, on the electronic properties of the complexes. Hence, as previously observed for **1**,^32^ the band at ∼320 nm is assigned to a π → π* transition of the coumarin chromophoric unit, i.e., to intraligand electronic transitions.^43−46^ This is corroborated from TDDFT theoretical calculations, which indicate that the relevant electronic transitions are located in the coumarin part of the molecule (see below). By changing the solvent’s polarity, here mirrored from the dielectric constant ε, the absorption spectral profile and maxima are found to be identical, as illustrated by the behavior in 2-methyltetrahydrofuran (2-MeTHF; ε = 7.58) and acetonitrile (MeCN; ε =37.5) (Figure 1 and Table 1).

The emission spectra show a broad emission band with maxima at 375–400 nm, again associated with the emission of **1**.^43−45^ Excitation spectra, obtained by collection at the emission wavelength maxima (see the vertical dashed lines in Figure 1), match the absorption of **1**, indicative of this core structure being in the origin of the emission. From the summarized spectral data (Table 1) of **1**, **1.1**, **1.2**, and **1.3**, it can be seen that their absorption and emission spectra are identical, thus showing that the spectral properties are basically those of the alkynylcoumarin percursor **1**.

In the solid state (thin films), a broader absorption with a ∼8–13 nm red shift of the absorption maxima is observed upon comparison with the spectra in solution (Figure 1). In general, similar spectroscopic features are observed in different solvents. Additionally, the fluorescence quantum yields (ϕ_F_) show that the compounds weakly fluoresce in a solution of good solvents, with values ranging from 1 to 3%, becoming moderately emissive in the solid state (thin films) with values of 5–12% (Table 1).

Upon a change from the solution to the solid state, broadening of the absorption band and the red shift of both the absorption and emission maxima are indicative of emissive aggregates in thin films with enhancement of the fluorescence quantum yield (AIE effect).

**Figure 2:**
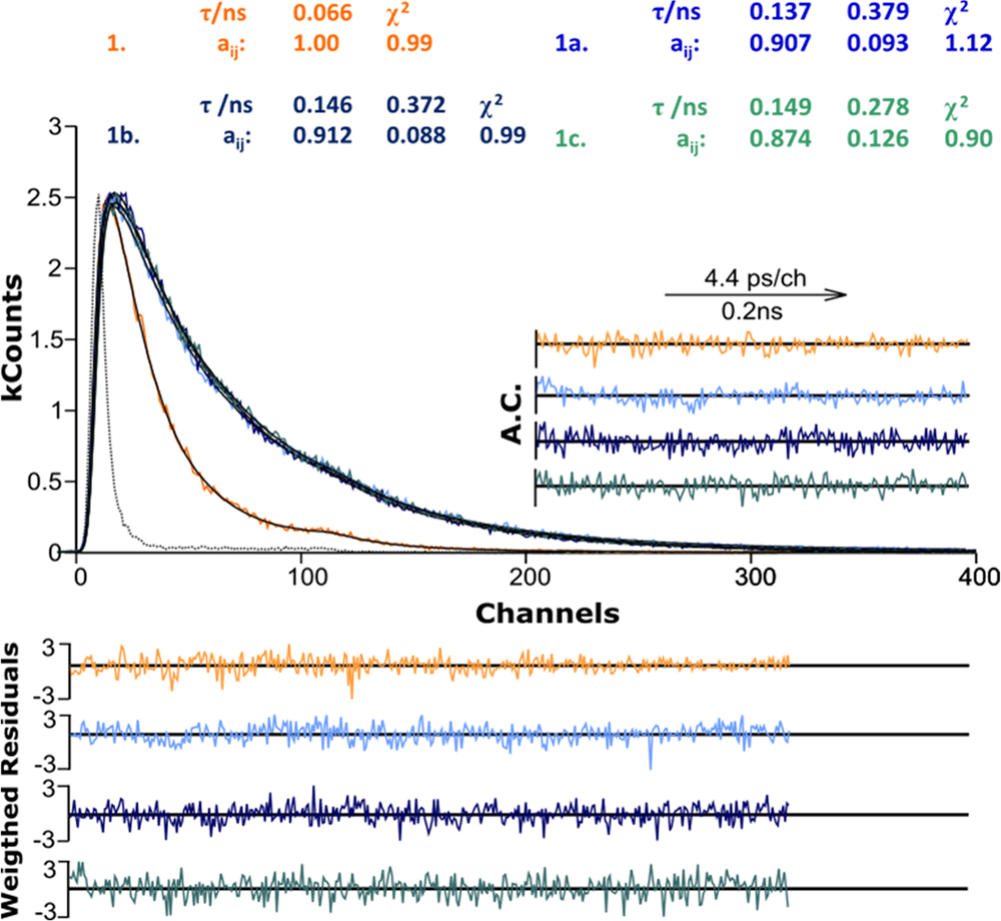
Fluorescence decays for the gold(I) complexes (**1.1**, **1.2**, and **1.3**) and **1** in dimethylformamide at *T* = 293 K with λ_exc_ = 268 nm and λ_em_ = 375 nm. The quality of the analysis is judged by the presentation of the weighted residuals, autocorrelation functions (A.C.), and χ^2^ values. The decay with the black dashed line is the IRF obtained with a scatter solution (see the Experimental Section).

Time-resolved fluorescence studies were further conducted to gain deeper insight into deactivation of the first singlet excited state of the studied compounds in solution (Figure 2). The fluorescence decays were analyzed with sums of exponentials, according to eq 1,
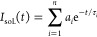
1where τ_*i*_ are the decay times and *a*_*i*_ are the preexponential factors that represent the contribution of each exponential term for *t* = 0. The fractional contribution of each species (*C*_*i*_) was determined using eq 2,^47^
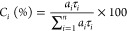
2with *n* the number of exponential terms, *a*_*i*_ the contribution of each of the exponential terms for *t* = 0, and τ_*i*_ the respective decay time. The data are summarized in Table S1 and Figure 2. The fluorescence decays of **1** were fitted to a single-exponential decay (with values ranging from 0.017 to 0.066 ns depending on the solvent), whereas that for the gold(I) complexes, in all solvents, the decays were found to be biexponential, with decay time values of 0.042–0.149 ns (τ_1_) and 0.116–0.430 ns (τ_2_). Furthermore, the amplitude (normalized preexponential factor) associated with the shorter decay time (*a*_*i*1_) is, in all solvents, found with the major contribution (Table S1). The second component, associated with the longer decay time (τ_2_) and with a smaller contribution (% *C*_2_), is, as will be rationalized in detail, also with the TDDFT data, with simulated structures of dimeric species (see the next section), attributed to the presence of ground-state aggregates.

Because the chromophoric and fluorogenic units in the complexes is the coumarin **1** ligand, the absence of even vestigial amounts of this compound in solutions of **1.1**, **1.2**, and **1.3** was indicated by the clearly different nature of the fluorescence decays. Indeed, besides the fact that for **1** the decay is single-exponential, in contrast with the gold(I) complexes, where it is double-exponential, the shorter component, τ_1_, is clearly much shorter in the precursor ligand **1**, 66 ps, than in **1.1**, **1.2**, and **1.3**, with ∼140 ps (Figure 2).

**Figure 3:**
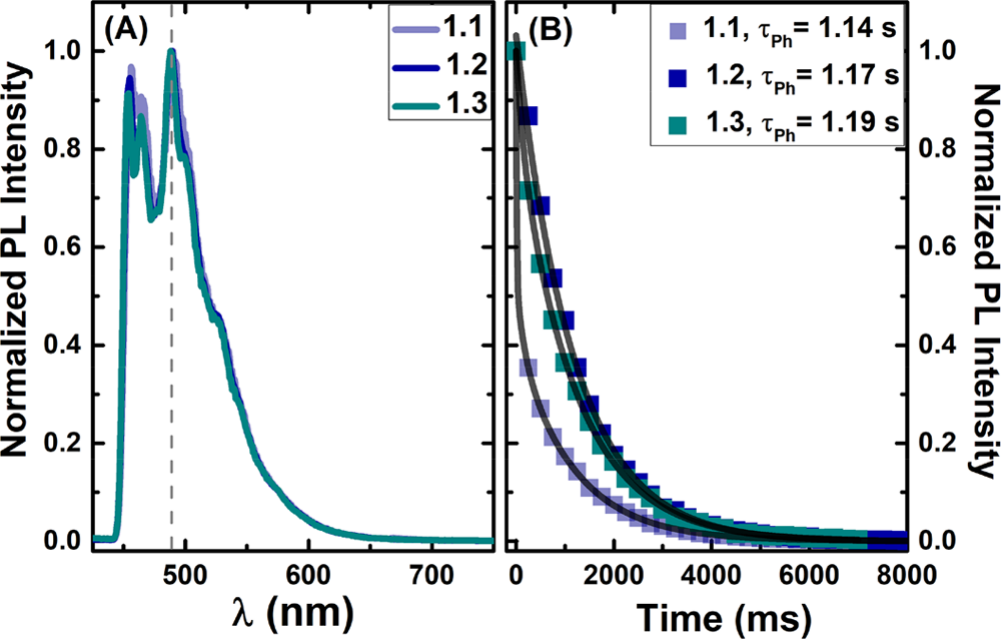
(A) Normalized phosphorescence emission spectra (λ_exc_ = 320 nm; DAF = 0.5 ms) and (B) decays (λ_em_ = 490 nm) for the gold(I) complexes **1.1**, **1.2**, and **1.3** in 2-MeTHF at *T* = 77 K.

Phosphorescence emission spectra and lifetimes for the three gold(I) complexes were obtained in 2-MeTHF at low temperature (77 K; Figure 3). The spectra were obtained with a pulsed xenon lamp and delays after the flash (DAF) of 0.0 and 0.5 ms. The assignment of the phosphorescence emission band was carried out with DAF = 0.5 ms, which is also accompanied by the appearance of a red-shifted emission band when it is compared to the characteristic fluorescence band in 2-MeTHF (Figure S12). The phosphorescence emission bands of the different gold(I) complexes are found to be independent of the excitation wavelength, fully overlapped, displaying a vibronically structured band centered at ∼490 nm (Figure 3 A), and are attributed to a ^3^π,π* state localized on the coumarin chromophoric unit.^32^ From the large Stokes shift observed and the long excited-state phosphorescence lifetimes (∼1.2 s; Figure 3 B), the triplet state has ^3^π,π* character, with high phosphorescence quantum yields (Table 2).

**Table 2:** Photophysical Data Including Quantum Yields (Fluorescence ϕ_F_, Phosphorescence τ_Ph_, and Singlet Oxygen Sensitization Fluorescence ϕ_Δ_) and Phosphorescence Lifetimes (τ_Ph_) Obtained in MeCN at 293 K and in 2-MeTHF at Low Temperature (77 K) for **1.1**, **1.2**, **1.3**, and **1**

	77 K				
	ϕ_F_	ϕ_Ph_	ϕ_F_ + ϕ_Ph_	τ_Ph_ (s)	ϕ_Δ_
**1**^a^	0.677	0.198	0.875	1.06	
**1.1**	0.426	0.269	0.695	1.14	0.021
**1.2**	0.490	0.172	0.662	1.17	0.022
**1.3**	0.466	0.193	0.659	1.19	0.029

a For **1** in MeCN and 2-MeTHF, the data are from ref (32).

The efficiency of singlet oxygen sensitization was obtained by measurement of the O_2_ phosphorescence emission (at 1270 nm) in aerated MeCN solutions, resulting in triplet energy transfer from **1.1**, **1.2**, and **1.3** to molecular singlet oxygen. The obtained values of the singlet oxygen sensitization quantum yields (ϕ_Δ_) were found to vary between 0.021 and 0.029 (Table 2), thus showing no appreciable changes among them. From the data, it can be concluded that the introduction of different phosphanes (DPPM, DPPP, and DPPA) and two gold(I) atoms does not have a significant impact on the singlet oxygen sensitization efficiency. Nevertheless, the presence of gold(I) is relevant for populating T_1_. Indeed, singlet oxygen generation could not be detected for **1**—therefore with nonsignificant population of the triplet state at room temperature—despite the ∼20% yield for phosphorescence (Table 1). Remember that ϕ_Δ_ is obtained at 293 K but ϕ_Ph_ at 77 K. By a comparison of previously investigated alkynylcoumarin gold(I) complexes with PTA and DAPTA phosphane linkers, with the 3-chlorocoumarin ligand derivative, ϕ_Δ_ values of 0.12 and 0.18 were obtained, making them good singlet oxygen sensitizers,^32^ the herein investigated gold complexes all display much lower values, likely because of the fact that a more efficient radiative deactivation channel is now present (Table 2).

From the overall photophysical data, some relevant aspects should be highlighted at this stage: (i) similar ϕ_F_ values (0.010–0.016) were observed in the presence and absence of oxygen; (ii) low quantum singlet oxygen sensitization was observed for **1.1**, **1.2**, and **1.3**, while no singlet oxygen sensitization effect was detected for **1**; (iii) the total emission, resulting from the fluorescence and phosphorescence quantum yields ϕ_F_ + ϕ_Ph_ in Table 2 is, at 77 K, high and higher than 66%, thus showing that the radiative processes dominate, at low temperatures, the deactivation of the excited state. Furthermore, at 77 K, the ratio ϕ_Ph_/ϕ_F_ decreases from **1** (ϕ_Ph_/ϕ_F_ = 3.4) to **1.1** (ϕ_Ph_/ϕ_F_ = 1.6) to **1.2** (ϕ_Ph_/ϕ_F_ = 2.8) to **1.3** (ϕ_Ph_/ϕ_F_ = 2.4), mirroring the contribution of the heavy atom effect, which favors the intersystem crossing quantum yield (increase of the SOC contribution due to the heavy atom effect promoted by the gold atom) and decreases ϕ_F_ in the gold(I) complexes (increase in the population of T_1_). This effect is particularly notorious with the phosphorescence value of **1.1**, which is higher than all of the others, indicating not only a more effective SOC due to the aurophilic effect, as predicted from TDDFT studies, but also a more efficient radiative deactivation of **1.1**.

#### AIE Studies

**Figure 4:**
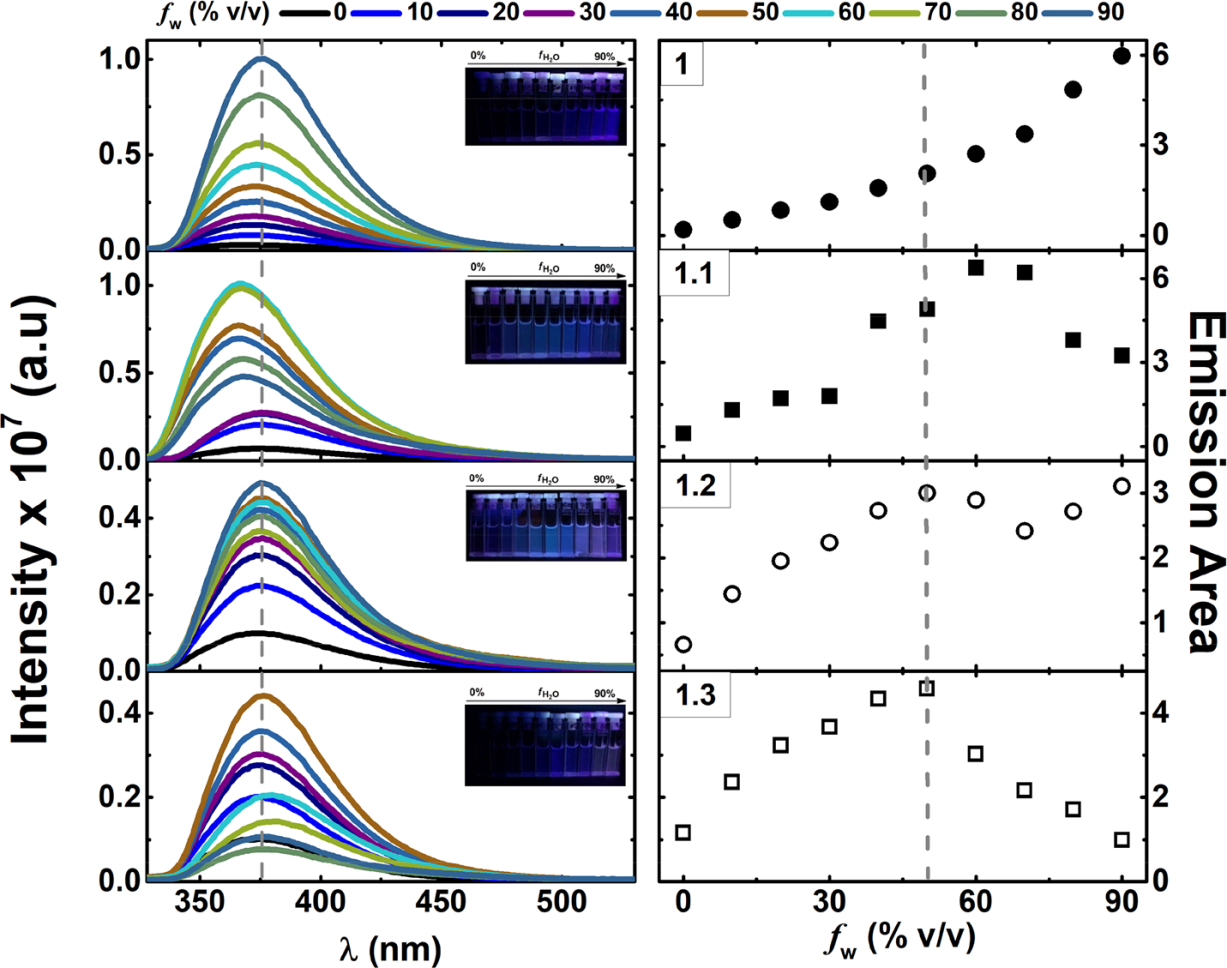
Fluorescence emission spectra (left panel) with pictures obtained under UV irradiation (with λ_exc_ = 254 nm) and correlation of the emission area (right panel) with increasing water fractions (*f*_w_) in MeCN/water mixtures for the three alkynylcoumarin dinuclear gold(I) complexes **1.1**, **1.2**, and **1.3** and the ligand **1**.

AIE occurrence in the gold(I) complexes **1.1**, **1.2**, and **1.3** and model compound **1** was investigated in MeCN/water mixtures (*f*_w_ is the volume percentage of water in MeCN/water mixtures). Figure 4 (left panel) shows the fluorescence emission spectra of **1.1**, **1.2**, **1.3**, and **1** in MeCN/water mixtures. The absorption spectra in MeCN/water mixtures are represented in Figure S13.

In MeCN/water mixtures, the fluorescence emission is seen to be clearly enhanced with the mixtures containing a high fraction of water. With an increase of the water percentage in the mixture, there is a continuing increase in the total fluorescence quantum yield up to water fraction values of 50% (**1.2** and **1.3**) and 60% (**1.1**). Moreover, in the case of **1.1**, a small blue shift is also observed in the emission spectra for the mixtures with a 40–90% water content. Indeed, the gradual addition of water to the MeCN solutions increases the solvent polarity and medium viscosity while decreasing the solvation power of the solution. Consequently, total emission is ruled out by the solvent, with ϕ_F_ increasing in MeCN (**1.1**, 0.010; **1.2**, 0.011; **1.3**, 0.012) to the 20:80 MeCN/water (% v/v) mixture (**1.1**, 0.089; **1.2**, 0.140; **1.3**, 0.119), i.e., an increment of 5–10 times depending on the compound and solvent mixture. With **1**, the increase in the fluorescence intensity with the water fraction is different from the pattern found for **1.1**, **1.2**, and **1.3** and not due to the AIE effect (Figure 4). Indeed, DFT and TDDFT calculations indicate a close proximity between, and mixing of, the two lowest-lying S_1_ (π,π*) and S_2_ (with an n,π* contribution) for **1**. The increase of the solvent polarity raises the energetic gap relative to the *S*_1_ (π,π*) and *S*_2_ (n,π*) states, thus decreasing the mixing of these states with an increase of the more polar solvent water in MeCN/water mixtures (with increasing *f*_w_), leading to S_1_ (π,π*) absent of a mixture with S_2_ of forbidden nature, being responsible for the increase of the resulting fluorescence emission.^48,49^

#### DLS Experiments in Mixtures of Good/Bad Solvents

**Figure 5:**
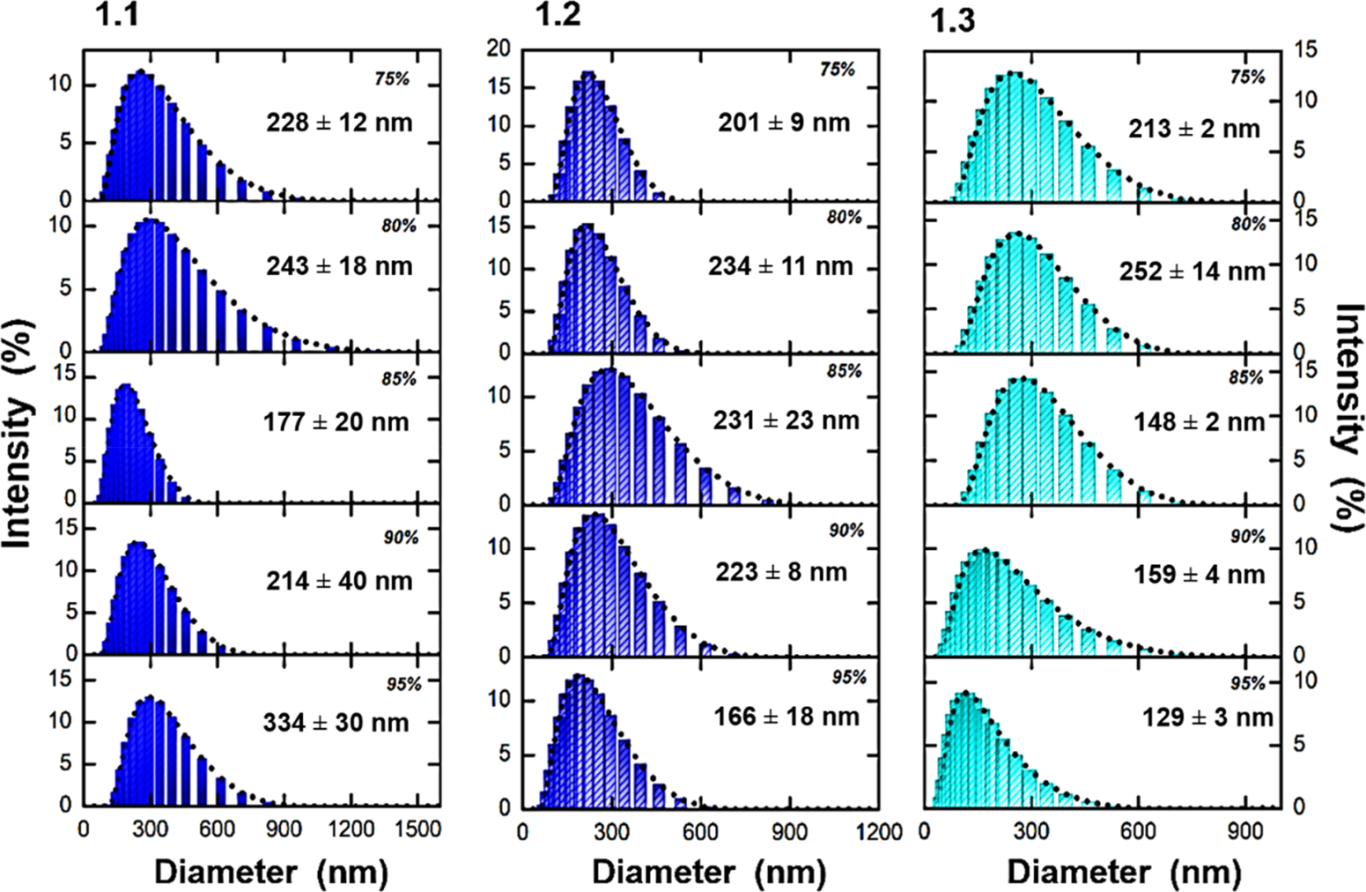
DLS particle size distribution curves obtained in MeCN/water mixtures (>75–95% H_2_O, v/v) for the gold(I) complexes **1.1**, **1.2**, and **1.3**. From top to bottom, *f*_w_ increases.

DLS experiments, performed in order to evaluate the formation and size of the aggregates in MeCN/water mixtures (Figure 5), corroborate their formation in MeCN/water mixtures and the decrease of the average size for *f*_w_ > 75%, in agreement with the observed decrease of the emission intensity (Figure 4). With **1**, no aggregates could be found.

#### Dependence with *f*_w_ of the Time-Resolved Fluorescence Data

The time-resolved fluorescence emission in MeCN/water mixtures (Figures S14–S19) is again shown, as in organic solvents, to be fitted to single-exponential (for **1**) and double-exponential (for **1.1**, **1.2**, and **1.3**) decay laws, with the decay time values increasing with *f*_w_ (Table S2 and Figures S14–S19). It is very interesting to see that, with **1.1**, **1.2**, and **1.3**, the increase of *f*_w_ leads to a decrease of the preexponential factor associated with the shorter decay time (τ_1_) and a concomitant increase of the preexponential factor associated with the longer component (τ_2_) (Figures S15, S17, and S19). The increase in the τ_2_ contribution (as seen by % *C*_2_) is also associated with an increase of the fluorescence quantum yield (Figure 4). Moreover, aggregates are already present in the ground state, which is particularly evident with excitation at 268 nm (Table S1). The second decay component is consequently assigned to the emissive aggregates and, thus, the shortest (τ_1_) decay time to the emission of monomeric (isolated) species. The monomer has lifetimes varying from 0.076 to 0.257 ns, whereas the lifetimes of the aggregates vary from 0.204 to 0.792 ns. Moreover, the preexponential (*a*_*i*2_) value, associated with the aggregates, increases concomitantly with *f*_w_. This can also be correlated with the DLS experiments, demonstrating the presence of a higher number of fluorescent aggregates at high *f*_w_ values (Figures 5 and S14–S19). This is further rationalized from TDDFT electronic calculations, namely, on the type of interaction established between the molecules (see the next section).

#### TDDFT Theoretical Studies

Theoretical DFT studies were performed in order to rationalize and better understand the experimental data, in particular the electronic properties of **1**, **1.1**, **1.2**, and **1.3**. The ground-state-optimized geometry structures were calculated together with the relevant highest occupied and lowest unoccupied molecular orbital energy levels and the electronic transitions (obtained by TDDFT) using the same level of theory, DFT//LC-BPBE(ω=0.2)/SBKJC (Stevens–Bash–Krauss–Jasien–Cundari). These results were compared to the experimental data. Frequency analyses for each compound were also computed and no imaginary frequencies were observed, which indicates that the structure of each one of the molecules corresponds, at least, to a local minimum on the potential energy surface (PES).

**Figure 6:**
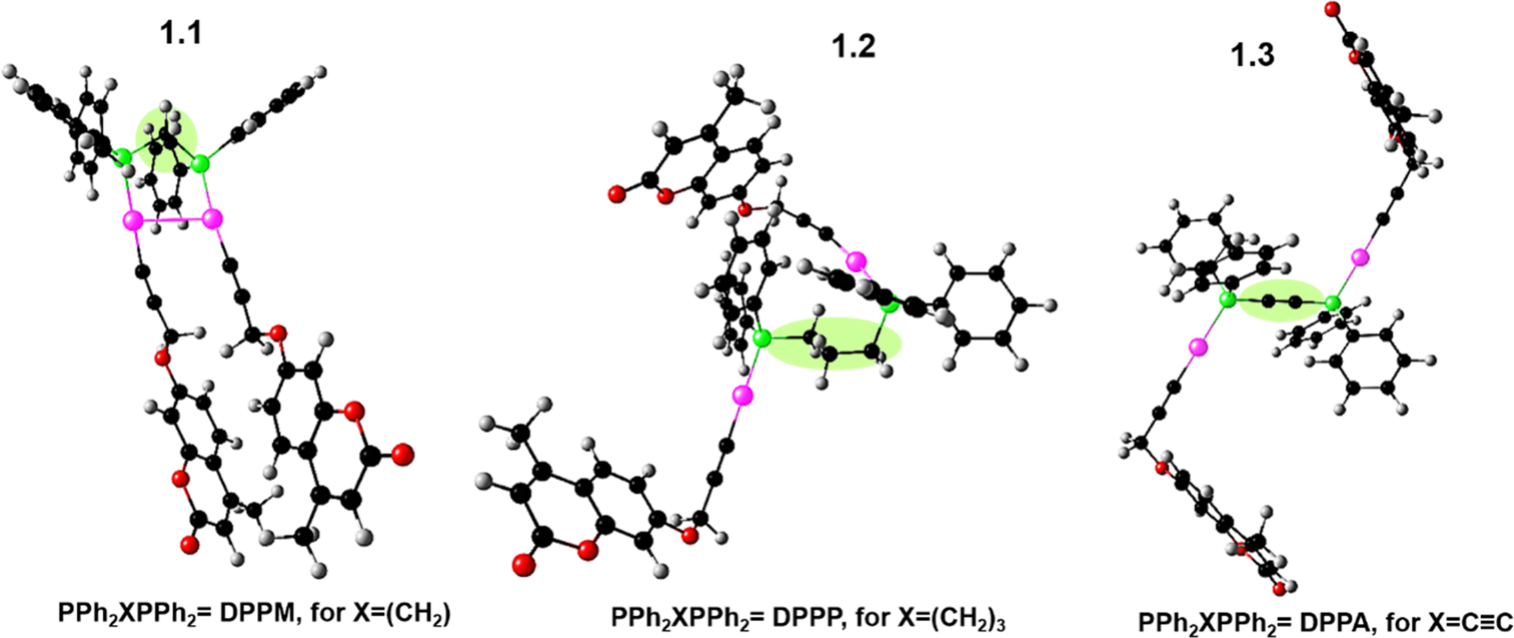
Energetically more favorable molecular structures, obtained from TDDFT calculations, for **1.1** (syn), **1.2** (anti), and **1.3** (anti).

Model compounds, with phosphane phenyl rings replaced by hydrogen atoms, were used to probe different conformational structures of the dinuclear gold(I) complexes **1.1**, **1.2**, and **1.3**. For DPPM, the most common structure, reported from different literature studies, involves the syn conformation with the Au–P chromophores in parallel orientation. In contrast, with DPPP and DPPA, it is the anti conformation, with Au–P chromophores in antiparallel positions, which is the most energetically favorable and therefore the most likely to be observed.^19,50,51^ From simple structure representations, conformers with anti and syn configurations can be easily drawn and their energy calculated (Figure S20). Among the different investigated structures (many others not depicted in Figure S20 have been considered in the calculations; see Figures S21–S23) and after verification of which structure (and conformation) is more stable for each compound, the different possible molecular geometries were optimized by DFT calculations and the main transitions, predicted for both absorption and emission, were analyzed. Figure 6 shows the most energetically stable structural conformer for **1.1**, **1.2**, and **1.3**.

From all of the above and considering the spectral and structural characteristics of these conformers, the following aspects should be highlighted: (i) In DPPM, the syn conformation is found to be the most stable configuration, whereas for DPPP and DPPA, it is the anti conformation, with the Au–P chromophores in antiparallel positions to each other (Figures S21–S23). (ii) Structurally, compound **1.1** is expected to exhibit, in all solvents, aurophilic interactions. Indeed, from the data in Table S4, it is possible to observe that the bonding distances between the two gold atoms in compound **1.1**, considering different solvation environments, are on the order of 2.991–3.016 Å. These values suggest the formation of aurophilic interactions in all solvents. Furthermore, the results obtained for the binding distance Au^I^···Au^I^ are in good agreement with literature values.^24,25,51^ (iii) Two pairs of nearly degenerate absorption bands (located in each of the two chromophores) are predicted: an intense band, in the range 310–314 nm, and a much less intense band, in the range 278–288 nm (depending on the ligand and solvent; see Table S3 for further details). With all compounds, the more intense (strong) band is essentially considered to be a π → π* transition located in the coumarin core, whereas the less intense (weak) band depicts significant charge-transfer character from the gold ethylene to the coumarin chromophore (MLCT). Considering the experimental data and TDDFT calculations that predict this transition with low *f* values (*f* < 0.05; Table S3; the contribution of this MLCT band is almost negligible, with the exception of **1.1**, which is likely to be associated with the aurophilic effect involving the proximity of the gold atoms).^32,52^ (iv) Calculations predict an emission band at 360 nm slightly blue-shifted (∼16 nm) compared to the experimental values of 373–376 nm (in MeCN; Table S5). From the representative orbital contours for each of these two transitions/bands (Figures S24–S26) for **1.1**, **1.2**, and **1.3**, in vacuum, the nature of the MLCT and aurophilic interactions can be further visualized. Additionally, triplet states are predicted to emit in the ∼460 nm range, with a 30 nm difference relative to the experimental value (490 nm in 2-MeTHF; Table S5).

#### AIE Effect Probed by TDDFT: Rationalizing the Double-Exponential Decay in MeCN/Water Mixtures

Although there is almost total qualitative equivalence between the experimental data and computed values for the absorption and emission spectra, an explanation for the observed time-resolved fluorescence studies (single exponential for **1** and double exponential for the gold(I) complexes, in particular in MeCN/water mixtures) is still missing. As a result of the high energy difference between the different possible conformers, only a single conformer could be found at room temperature (Figure 6), therefore failing to rationalize the biexponential nature of the fluorescence decays as being the result of the presence of two stable conformers. The nature of the double-exponential decays will therefore be rationalized in the following paragraphs and essentially involve the formation of an emissive aggregate coexisting with a monomer.

**Figure 7:**
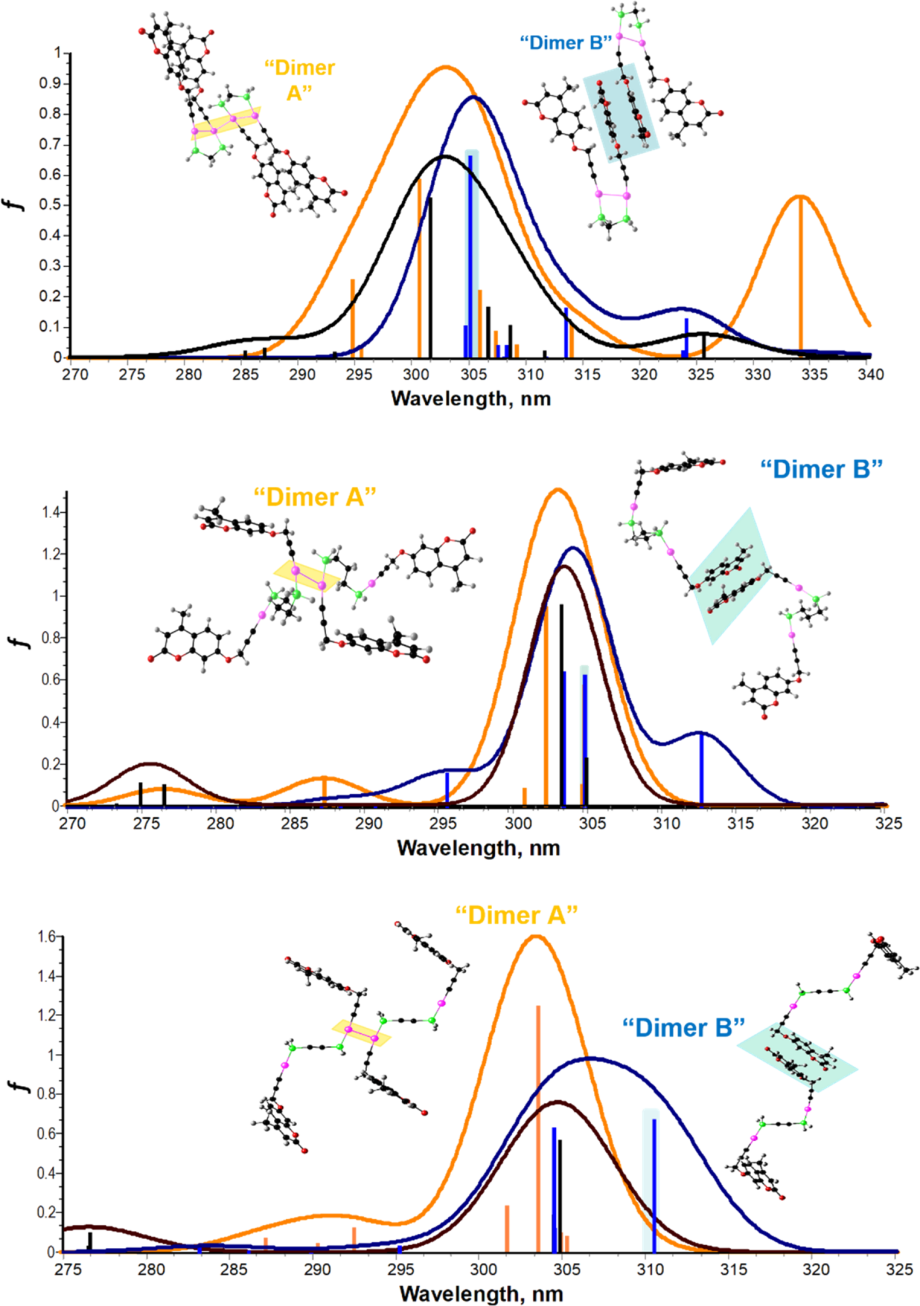
TDDFT absorption spectra of two *dimers* of **1.1**, **1.2**, and **1.3**, respectively, with different orientations and of the monomer and both *dimers*. Color legend: black, monomer; orange, “Dimer A”; blue, “Dimer B”.

As shown above, model compounds with phosphane phenyl rings were replaced by hydrogen atoms, and their geometry was optimized; the computed absorption and emission maxima showed no substantial deviations (less than 2 nm) from the gold(I) complexes (Figures S24–S26). These simpler models were used to probe the different dimer/aggregate conformational structures of the complexes (Figure 7). The analysis was followed by the use of the most stable conformer to promote aggregate, in this case the simpler dimer, formation. Two *dimers*, of **1.1**, **1.2**, and **1.3**, with distinct orientations were explored: one of the *dimers* is oriented by the {P–Au–C≡C} fragments in parallel (“Dimer A”), whereas the other structure is oriented with parallel coumarin ring moieties (“Dimer B”) (Figure 7). DFT electronic calculations on the nature of the excited states of these two *dimers* allow us to conclude that “Dimer B” should be considered as more suitable because there is a lowering of its energy in comparison with the monomeric species (isolated molecule; Figure 7).

This additional *dimer* (or aggregate species)—in addition to the monomer—now accounts for the observed biexponential decays. TDDFT-generated absorption spectra of the aggregate (in the present cases well accounted as a *dimer*, “Dimer B”; Figure 7) show that a new absorption band, ca. 5 nm (**1.1**), 10 nm (**1.2**), and 5 nm (**1.3**) red-shifted relative to the monomer band, is observed. This “Dimer B” band involves the contribution of the molecular orbitals located in the two stacked coumarins (resulting from π–π stacking involving two coumarin rings but from different monomer units; Figures S27–S29). Experimentally (Figure 1), this new band is observed as a shoulder of the main transition (π → π*), with molecular orbitals located in the terminal coumarin cores.

## Conclusions

Three propargyloxycoumarin diphosphane gold(I) complexes (**1.1**, **1.2**, and **1.3**), investigated in MeCN/water mixtures and in the solid state (thin film), have shown the AIE effect to be absent in the organic precursor, the propynyloxycoumarin ligand (**1**), by the absence of aggregates (from DLS) and by a single-exponential fluorescence decay, in contrast with the double-exponential decay found for **1.1**, **1.2**, and **1.3**. The overall behavior is rationalized with TDDFT calculations, leading to different favorable syn (**1.1**) and anti (**1.2** and **1.3**) conformers and the formation of an emissive aggregate “dimer” with an antiparallel orientation of the coumarin rings (chromophoric unit). This is further cosubstantiated from DLS measurements, showing an increase of the molecular volume resulting from π–π stacking between the two coumarin rings and from time-resolved fluorescence data, where aggregates coexist with monomer species with different decay times. The presence of the two gold atoms, together with the change in size and flexibility of the different phosphanes, does not determine the dominant interaction responsible for the aggregate emission. Larger aggregates can be built from the dimer structure of the different gold(I) complexes. However, the chromophoric unit, responsible for the absorption and emission properties, should be considered to be that of the *dimer*, thus showing that larger aggregates essentially behave as if they are this species.

## Experimental Section

### General Procedures

For the synthetic procedures, all operations were performed under prepurified dinitrogen using standard Schlenk techniques. Solvents were distilled from the appropriate drying agents. The commercial reagents bis(diphenylphospino)methane (DPPM), 1,3-bis(diphenylphospino)propane (DPPP), and bis(diphenylphospino)acetylene (DPPA) were used as received from Sigma-Aldrich. Literature methods were used to prepare the synthesis of 4-methyl-7-(prop-2-in-1-yloxy)-1-benzopyran-2-one.^52^

The solvents were of spectroscopic or equivalent grade and were used as received. MeCN/water solutions were prepared using deionized water (18.2 MΩ·cm at 25 °C; Milli-Q, Millipore). For the photophysical experiments, removal of the oxygen dissolved in the solutions was performed by bubbling the solutions with a stream of argon or nitrogen for approximately 20–30 min in a home-built quartz cuvette described elsewhere.^53^ All measured solutions were freshly prepared (within 1 day).

### Physical Measurements

IR spectra were recorded with a Nicolet FT-IR 520 spectrophotometer. ^1^H NMR [δ(TMS) = 0.0 ppm; TMS = tetramethylsilane] and ^31^P NMR [δ(85% H_3_PO_4_) = 0.0 ppm] spectra were obtained on Varian Mercury 400 and Bruker 400 (Universitat de Barcelona) spectrometers. Positive-ion-mode electrospray ionization mass spectrometry [ESI-MS(+)] spectra were recorded on a Fisons VG Quatro spectrometer. Chemical shifts are given in δ (ppm) relative to TMS (^1^H) or CDCl_3_ (^31^P), and coupling constants *J* are given in hertz. The multiplicity is expressed as s (singlet), d (doublet), and m (multiplet). Numbering schemes for the compounds characterized are displayed in Scheme 1. Absorption spectra were obtained in a 5 or 10 mm quartz cuvette in MeCN on a Shimadzu UV-2600 spectrophotometer. Fluorescence emission spectra were obtained with a fluorescence quartz cuvette of 5 or 10 mm path length using a Horiba-Jobin-Vonn Fluorolog 3.22 or a Fluoromax spectrometer. Phosphorescence spectra and decays were obtained with the D1934 unit of a Horiba-Jobin-Vonn Fluorolog 3.22 spectrometer using a pulsed xenon lamp. Fluorescence and phosphorescence spectra were corrected for the wavelength response of the system.

### Determination of the Emission Quantum Yields

The fluorescence quantum yields for the solid and solution samples were obtained by an absolute method using a Hamamatsu Quantaurus QY model C11437 absolute photoluminescence quantum yield spectrometer (integration sphere). The absorption of the solutions was kept under 0.1 at the excitation wavelength to avoid the inner filter effect.^54^ For the solid-state samples (thin films), the fluorescence quantum yields were obtained with the same Hamamatsu Quantaurus QY integration sphere.

### Determination of the Emission Singlet Oxygen Quantum Yields

The phosphorescence of singlet oxygen at room temperature was detected at an emission wavelength of 1270 nm with a Horiba-Jobin-Ivon SPEX Fluorolog 3.22 spectrofluorimeter using a Hamamatsu R5509-42 photomultiplier cooled with liquid nitrogen. A Schott RG1000 filter, to eliminate all of the first-harmonic contributions of the sensitizer emission in the region below 850 m, from the IR signal, was used. The singlet oxygen formation quantum yield was subsequently determined by direct measurement of the phosphorescence signal at 1270 nm, Emission_1270 nm_ (in eq 3), following irradiation of the aerated solution of the samples in MeCN. The standard used was 1*H*-phenal-1-one in MeCN (ϕ_Δ_= 0.98),^55^ and using eq 3, the singlet oxygen formation quantum yield of our compounds was obtained.
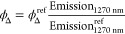
3where ϕ_Δ_^ref^ stands for the singlet oxygen formation quantum yield of the reference compound.

### Time-Resolved Fluorescence Measurements

Fluorescence decays were obtained with a home-built picosecond time-correlated single-photon-counting (TCSPC) apparatus, described in detail elsewhere.^56^ The equipment can be briefly described as follows: excitation was obtained from a picosecond Spectra Physics mode-lock Tsunami laser (Ti:sapphire) model 3950 (80 MHz repetition rate; tuning range 700–1000 nm), which was pumped by a continuous-wave solid-state Millennia Pro-10s laser (532 nm), and the third harmonic with a wavelength of 275 nm was generated with a Spectra Physics GWU-23PS component. An Oriel Cornerstone 260 monochromator and a Hamamatsu multichannel photomultiplier (R3809U-50) were used for emission wavelength selection and signal detection. The signal acquisition and data processing were performed with a Becker & Hickl SPC-630 TCSPC module. Fluorescence decays and instrumental response functions (IRFs) were collected using 1024 or 4096 channels in time scales varying from 3.26 to 6.4 ps/channel scale, until 5000 counts were reached. The full width at half-maximum (fwhm) of the IRF was 25 ps. Deconvolution of the fluorescence decay curves was performed using the modulating function method in the *SAND*(57) program by Striker et al., which further allows a value of ca. 10% of the fwhm (∼2 ps) as the time resolution of the equipment with this excitation source.

### DLS Measurements

DLS studies were performed using a Zetasizer Nano ZS (Malvem Panalytical). The size distribution of the aggregates was measured in a 10 mm quartz cuvette with a final volume of 1 mL, at 20 °C, in three consecutive runs of the same sample. The refractive index and viscosity of the MeCN/water mixtures were determined in advance at the experiment temperature and seen to be in agreement with those found in the literature for different reported temperatures.^58^

### Sample Preparation

A 3 mL stock solution of all compounds in MeCN with an absorption of 0.5–0.6 (at 320 nm) in a 10 mm quartz cuvette was prepared. An aliquot (200 μL) of the stock solution was transferred to a 2 mL volumetric flask. After the appropriate amount of MeCN was added, water was added to furnish mixtures with different water fractions (*f*_w_ = 0–90% by volume) with the same compound concentrations. The photophysical studies of the resultant mixtures were performed immediately after the sample preparation.

Thin films from the compounds were obtained using a desktop precision spin-coating system, model P6700 series from Speedline Technologies, as described elsewhere.^59^ Briefly described, thin films from the samples were obtained by the deposition of ca. 50 μL from a solution of the compounds into a circular sapphire substrate (10 mm diameter), followed by spin coating (2500 rpm) in a nitrogen-saturated atmosphere (2 psi). The solutions for spin coating were prepared by adding 2 mg of the samples to 200 μL of a chloroform solution containing 15 mg of Zeonex. Before the film deposition procedure was performed, the solutions were stirred overnight at room temperature.

### TDDFT Calculations

All theoretical calculations were of the DFT type and were carried out using *GAMESS-US*, version R3.^60^ A range-corrected LC-BPBE (ω = 0.20 au^–1^) functional, as implemented in *GAMESS-US*,^60^ was used in both ground- and excited-state calculations. TDDFT calculations, with similar functionals, were used to probe the excited-state PES. A solvent was included using the polarizable continuum model with the solvation model density to add corrections for cavitation, dispersion, and solvent structure. In TDDFT calculation of the Franck–Condon excitations, the dielectric constant of the solvent was split into a “bulk” component and a fast component, which is essentially the square of the refractive index. Under “adiabatic” conditions, only the static dielectric constant was used. DFT and TDDFT calculations, for location of the critical points, were carried out using SBKJC effective core potentials for nonvalence electrons, with a split-31G for valence electrons.^61−63^ The results obtained with the LC-BPBE(20) functional are essentially unscaled raw data from calculations; for the S_0_ → S_*n*_ transitions, a small correction, which results in the subtraction of 0.05 eV to account for the difference between the zero point and the first vibronic level, was considered. TDDFT calculations (using the same functional and basis set as those in the previous calculations) were performed for the resulting optimized geometries to predict the vertical electronic excitation energies. Molecular orbital contours were plotted using the *ChemCraft 1.7* program. The frequency analysis for each compound was also computed and did not yield any imaginary frequencies, indicating that the structure of each molecule corresponds to at least a local minimum on the PES.

### Synthesis of the Propynyloxycoumarin Ligand (**1**)

The organic alkynyl ligand was prepared by a method previously reported in the literature.^52^

### Synthesis of [Au(C≡C_13_H_9_O_3_)]_*n*_ (**1a**)

The organic alkynyl ligand **1** (51 mg, 0.238 mmol) was dissolved in dichloromethane (10 mL) and allowed to stir for 10 min. A sodium acetate base (49 mg, 0.595 mmol), previously dissolved in methanol, was added at the stoichiometry of 1:2.5. The solid AuCl(tht), in a 1:1 stoichiometry (72 mg, 0.225 mmol), was transferred to the reaction mixture which was allowed to stir for approximately 30 min. The product was filtered and dried under vacuum. A white solid was obtained in 80% yield.

IR (KBr, cm^–1^): 2022 (C≡C), 1716 (C=O).

### Synthesis of [Au{4-methyl-7-(prop-2-in-1-yloxy)-1-benzopyran-2-one} (DPPM)]_2_ (**1.1**)

Solid DPPM (15 mg, 0.04 mmol) was added to a suspension of **1a** (31 mg, 0.15 mmol) in dichloromethane (15 mL). After 60 min of stirring at room temperature, the resulting pale-yellow solution was concentrated (5 mL), and *n*-hexane (15 mL) was added to precipitate a pale-yellow solid, which was obtained in 56% yield (26 mg).

^1^H NMR (CDCl_3_): δ 7.56–7.47 (m, 8H, H_ortho_Ph), 7.43–7.30 (m, 14H, H_meta,para_Ph + 2O–C–C*H*–C*H*), 7.01–6.95 (m, 4H, 2O–C–CH–C*H* + O–C–C*H*–C), 6.06 (q, 2H, *J* = 21.0 Hz, CO–C*H*–C), 4.91 (s, 4H, 2C*H*_2_), 3.54 (t, *J* = 10.8 Hz, 2H), 2.34 (s, 6H, 2C*H*_3_). ^31^P{^1^H} NMR (CDCl_3_): δ 31.0. IR (KBr, cm^–1^): 2131 (C≡C), 1709 (C=O). HRESI-MS(+): *m*/*z* 1227.1474 ([M + Na]^+^, calcd *m*/*z* 1227.1523), 1243.1244 ([M + K]^+^, calcd *m*/*z* 1243.1263).

### Synthesis of [Au{4-methyl-7-(prop-2-in-1-yloxy)-1-benzopyran-2-one} (DPPP)]_2_ (**1.2**)

The same synthesis as that of **1.1** was used in the preparation of this compound, but DPPP (15 mg, 0.04 mmol) was used instead of DPPM. A pale-yellow solid was obtained in 66% yield (30 mg).

^1^H NMR (CDCl_3_): δ 7.65–7.62 (m, 8H, H_ortho_Ph), 7.60–7.39 (m, 14H, H_meta,para_Ph + 2O–C–C*H*–C*H*), 7.05–6.96 (m, 4H, 2O–C–CH–C*H* + O–C–C*H*–C), 6.11 (q, 2H, *J* = 21.5 Hz, CO–C*H*–C), 4.90 (s, 4H, 2C*H*_2_), 2.73 (m, 4H), 2.36 (s, 6H, 2C*H*_3_), 1.87 (m, 2H). ^31^P{^1^H NMR (CDCl_3_): δ 34.3. IR (KBr, cm^–1^): 2135 (C≡C), 1704 (C=O). HRESI-MS(+): *m*/*z* 1233.1971 ([M + H]^+^, calcd *m*/*z* 1233.2017), 1255.1862 ([M + Na]^+^, calcd *m*/*z* 1255.1836), 1271.1528 ([M + K]^+,^ calcd *m*/*z* 1271.1576), 2482.4192 ([2M + NH_4_]^+^, calcd *m*/*z* 2482.4227), 2487.3756 ([2M + Na]^+^, calcd *m*/*z* 2487.378).

### Synthesis of [Au{4-methyl-7-(prop-2-in-1-yloxy)-1-benzopyran-2-one} (DPPA)]_2_ (**1.3**)

The same synthesis as that of **1.1** was used in the preparation of this compound, but DPPA (15 mg, 0.04 mmol) was used instead of DPPM. A pale-yellow solid was obtained in 48% yield (23 mg).

^1^H NMR (CDCl_3_): δ 7.74–7.69 (m, 8H, H_ortho_Ph), 7.55–7.48 (m, 14H, H_meta,para_Ph + 2O–C–C*H*–C*H*), 7.06–6.96 (m, 4H, 2O–C–CH–C*H* + O–C–C*H*–C), 6.13 (q, 2H, *J* = 21.7 Hz, CO–C*H*–C), 4.91 (s, 4H, 2C*H*_2_), 2.39 (s, 6H, 2C*H*_3_). ^31^P{^1^H} NMR (CDCl_3_): 17.0. IR (KBr, cm^–1^): 2137 (C≡C), 1710 (C=O). HRESI-MS(+): *m*/*z* 1237.1323 ([M + Na]^+^, calcd *m*/*z* 1237.1367), 1253.1056 ([M + K]^+^, calcd *m*/*z* 1253.1106), 2446.326 ([2M + NH_4_]^+^, calcd *m*/*z* 2446.3288), 2451.281 ([2M + Na]^+^, calcd *m*/*z* 2451.2841).

## Supporting Information Available

Photoluminescence spectra of gold(I) complexes in MeCN/water mixtures, particle size distribution studies of gold(I) complexes in MeCN/water mixtures, ^1^H and ^31^P NMR and ESI-MS(+) spectra, and DFT/LC-BPBE(ω=0.2)/SBKJC-optimized ground-state geometries of the coumarin and dinuclear gold(I) complexes (PDF)
