# Supplementary material for: Aggregation-Induced Emission with Alkynylcoumarin Dinuclear Gold(I) Complexes: Photophysical, Dynamic Light Scattering, and Time-Dependent Density Functional Theory Studies
Source: Inorg Chem. 2022 Apr 27;61(18):6964–76. doi: 10.1021/acs.inorgchem.2c00366 (PMC9775461; doi:10.1021/acs.inorgchem.2c00366)
Supplement: Supplementary file 1 — ic2c00366_si_001.pdf [file ic2c00366_si_001.pdf]

Supplementary Information

Aggregation-Induced Emission with Alkynylcoumarin Dinuclear Gold(I) Complexes: Photophysical, Dynamic Light Scattering and TD-DFT Studies

Carla Cunha,[a] Andrea Pinto,[b] Adelino Galvão,[d] Laura Rodríguez [b,c*] and J. Sérgio Seixas de Melo,[a*]

[a]*University of Coimbra, Coimbra Chemistry Centre, Department of Chemistry, Rua Larga, Coimbra3004-535, Portugal. e-mail: sseixas@ci.uc.pt*

[b]*Departament de Química Inorgànica i Orgànica, Secció de Química Inorgànica, Universitat de Barcelona, Martí i Franquès 1-11, E-08028 Barcelona, Spain. e-mail: laura.rodriguez@qi.ub.es*

[c]*Institut de Nanociència i Nanotecnologia (IN2UB). Universitat de Barcelona, 08028 Barcelona, Spain*

[d]*Centro de Química Estrutural, Instituto Superior Técnico (IST), Universidade de Lisboa, Lisboa, Portugal*

Table of contents

Experimental Section 3

Figure S1. IR spectrum of the propynyloxycoumarin precursor (**1**, 4-methyl substituted coumarin). 3

Figure S2. IR spectrum of the $[Au(C\equiv C_{13}H_9O_3)]_n$ polymer (**1a**) 3

Figure S3. ^{1}H NMR spectrum of the compound **1.1** in $CDCl_3$. 4

Figure S4. ^{1}H NMR spectrum of the compound **1.2** in CDCl3. 4

Figure S5. ^{1}H NMR spectrum of the compound **1.3** in $CDCl_3$. 5

Figure S6. ^{31}P NMR spectrum of the compound **1.1** in $CDCl_3$. 5

Figure S7. ^{31}P NMR spectrum of the compound **1.2** in $CDCl_3$ 6

Figure S8. ^{31}P NMR spectrum of the compound **1.3** in $CDCl_3$. 6

Figure S9. ESI-MS(+) spectrum of the compound **1.1**. 7

Figure S10. ESI-MS(+) spectrum of the compound **1.2**. 7

Figure S11. ESI-MS(+) spectrum of the compound **1.3**. 8

Table S1. Time resolved fluorescence data 8

Figure S12. Normalized spectra of gold(I) complexes in 2-MeTHF. 9

Figure S13. Absorption spectra with increasing water fractions (fw) in MeCN: water mixtures 9

Table S2. Time resolved fluorescence data 10

Figure S14. Room-temperature fluorescence decays for **1.1** in different fraction water 10

Figure S15. Fluorescence decay parameters obtained for compound **1.1** in MeCN: water mixture 11

Figure S16. Room-temperature fluorescence decays for **1.2** in different fraction water 11

Figure S17. Fluorescence decay parameters obtained for compound **1.2** in MeCN: water mixture 12

Figure S18. Room-temperature fluorescence decays for **1.3** in different fraction water 12

Figure S19. Fluorescence decay parameters obtained for compound **1.3** in MeCN: water mixture 13

Figure S20. Simplified scheme of the representation of the conformational possibilities with syn vs. anti conformation 13

Figure S21. Model compounds to probe the conformational space (with syn and anti conformation) of **1.1** 14

Figure S22. Model compounds to probe the conformational space (with syn and anti conformation) of **1.2**. 14

Figure S23. Model compounds to probe the conformational space (with syn- and anti-conformation) of **1.3** 15

Table S3. Absorption maximum experimental (Exp.) and theoretical (Calc.) values, in different solvents, for organic ligand (**1**) and gold(I) complexes. 15

Table S4. Distance Au(I)···Au(I) (in Å), in the different solvents 16

Figure S24. TD-DFT absorption spectra of the compound **1.1** 16

Figure S25. TD-DFT absorption spectra of the compound **1.2** 17

Figure S26. TD-DFT absorption spectra of the compound **1.3** 18

Table S5. Calculated energies for lowest singlet and triplet states S_1 and T_1 19

Figure S27. Orbital contours of the HOMO and LUMO for complex **1.1** – "Dimer B" (aggregate). 19

Figure S28. Orbital contours of the HOMO and LUMO for complex **1.2** – "Dimer B" (aggregate). 20

Figure S29. Orbital contours of the HOMO and LUMO for complex **1.3** – "Dimer B" (aggregate). 20

Experimental Section

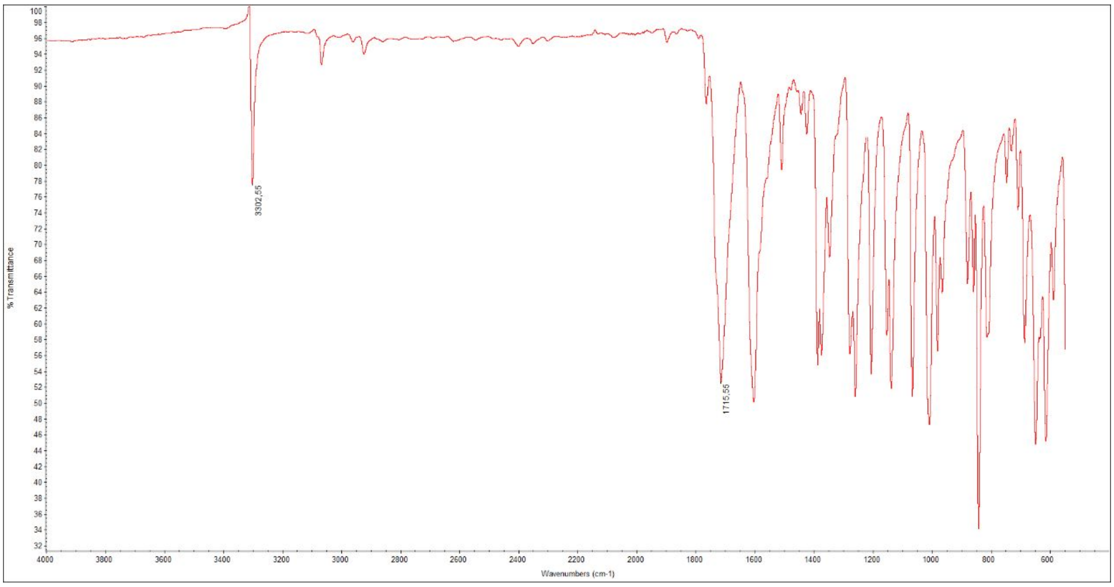

Figure S1. IR spectrum of the propynyloxycoumarin precursor (**1**, 4-methyl substituted coumarin).

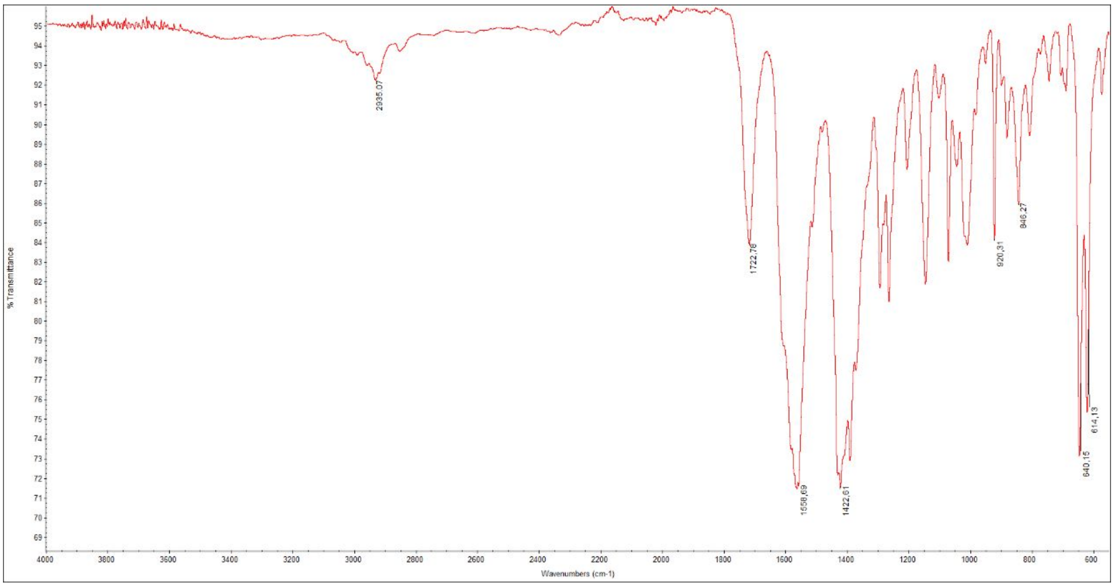

Figure S2. IR spectrum of the $[Au(C\equiv C_{13}H_9O_3)]_n$ polymer (**1a**).

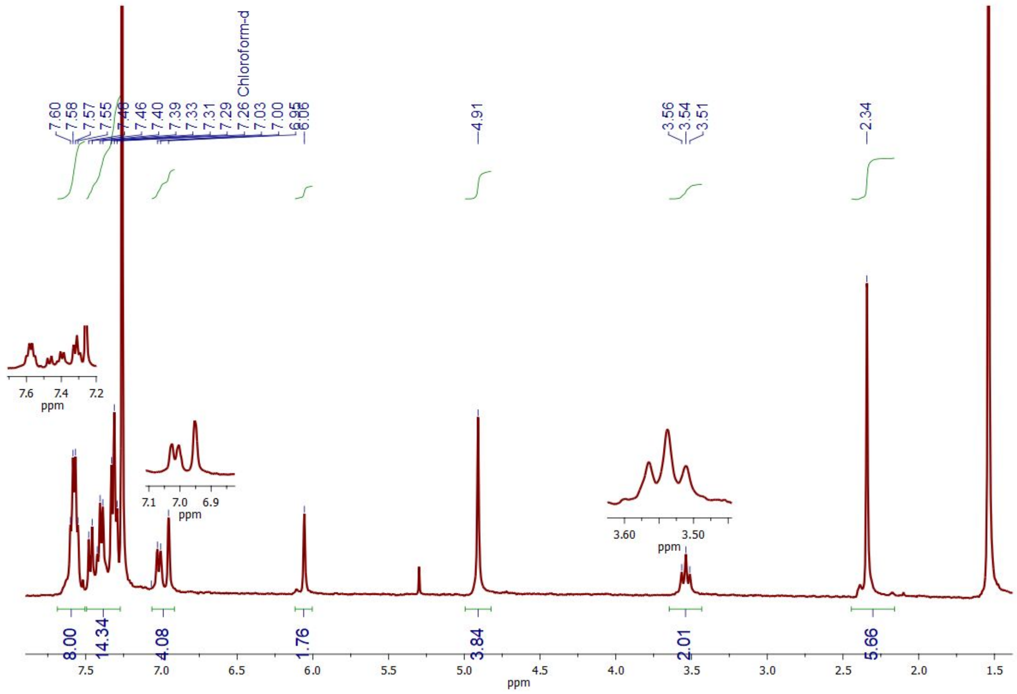

Figure S3. ^{1}H NMR spectrum of the compound **1.1** in $CDCl_3$.

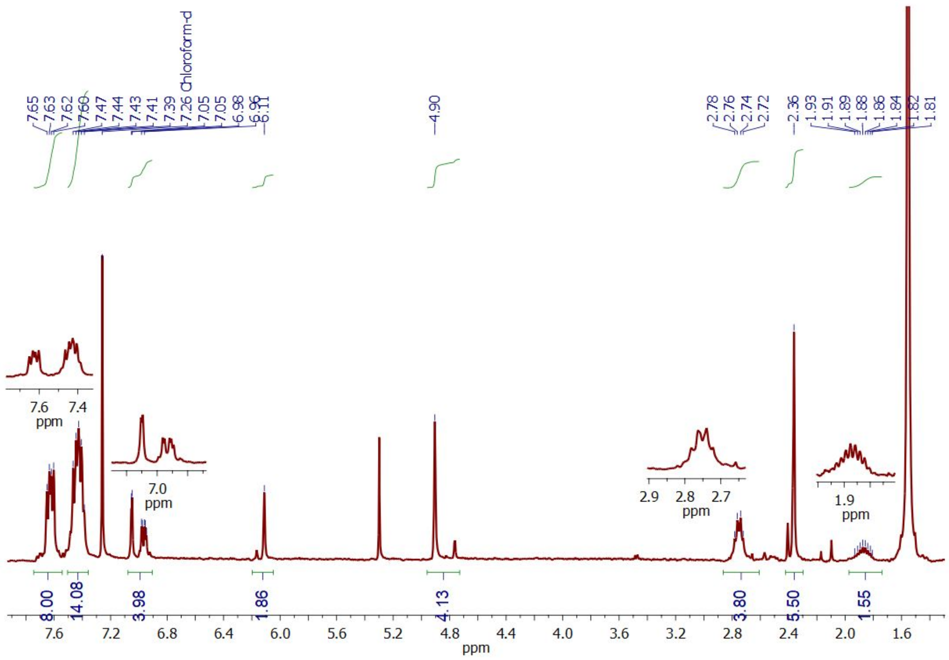

Figure S4. ^{1}H NMR spectrum of the compound **1.2** in CDCl3.

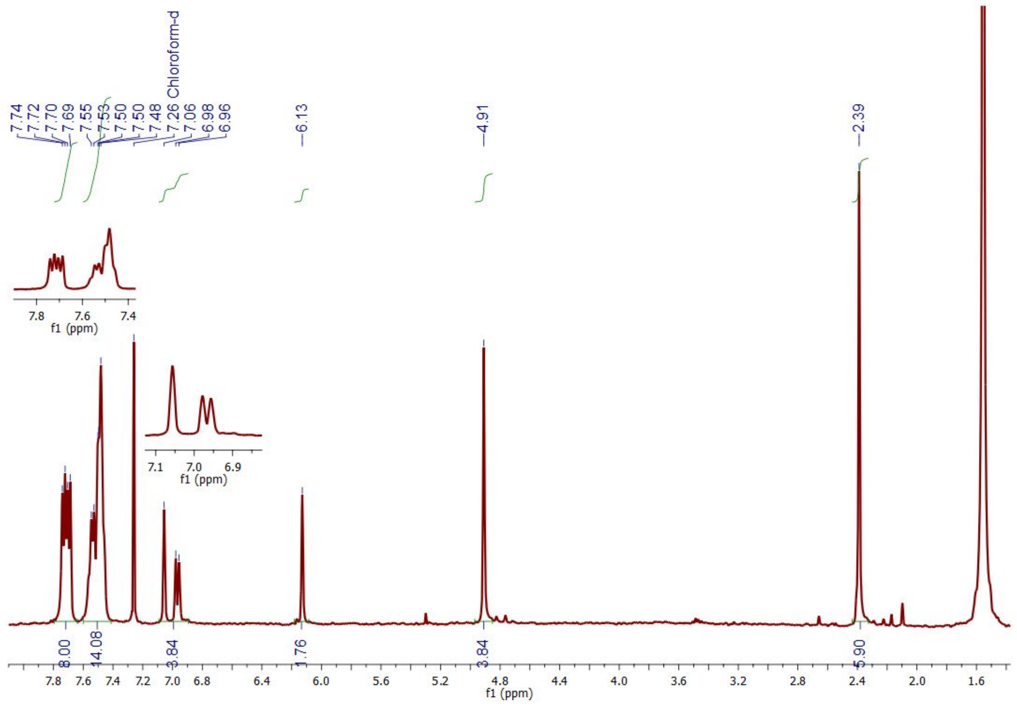

Figure S5. ^{1}H NMR spectrum of the compound **1.3** in $CDCl_3$.

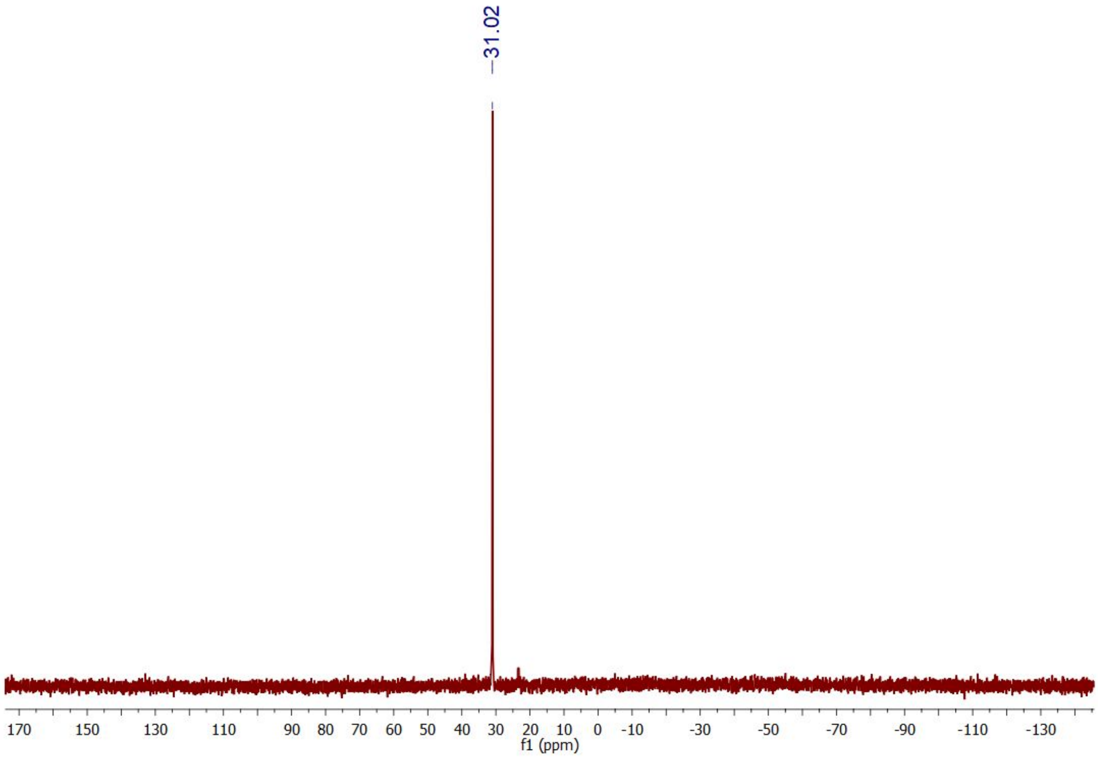

Figure S6. ^{31}P NMR spectrum of the compound **1.1** in $CDCl_3$.

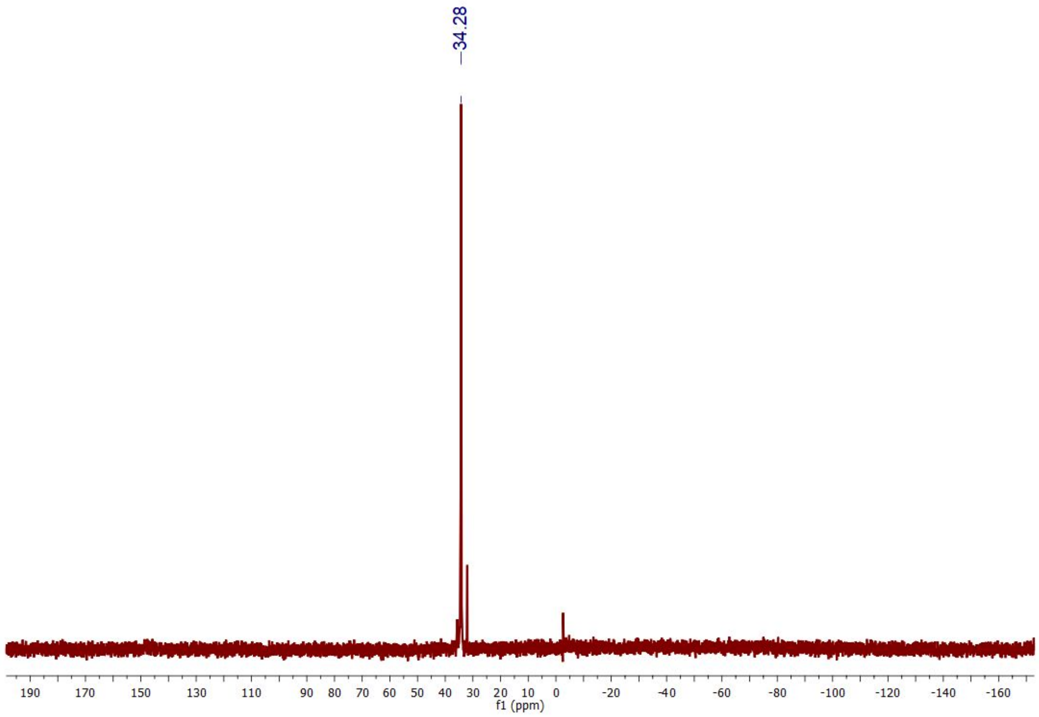

Figure S7. ^{31}P NMR spectrum of the compound **1.2** in $CDCl_3$.

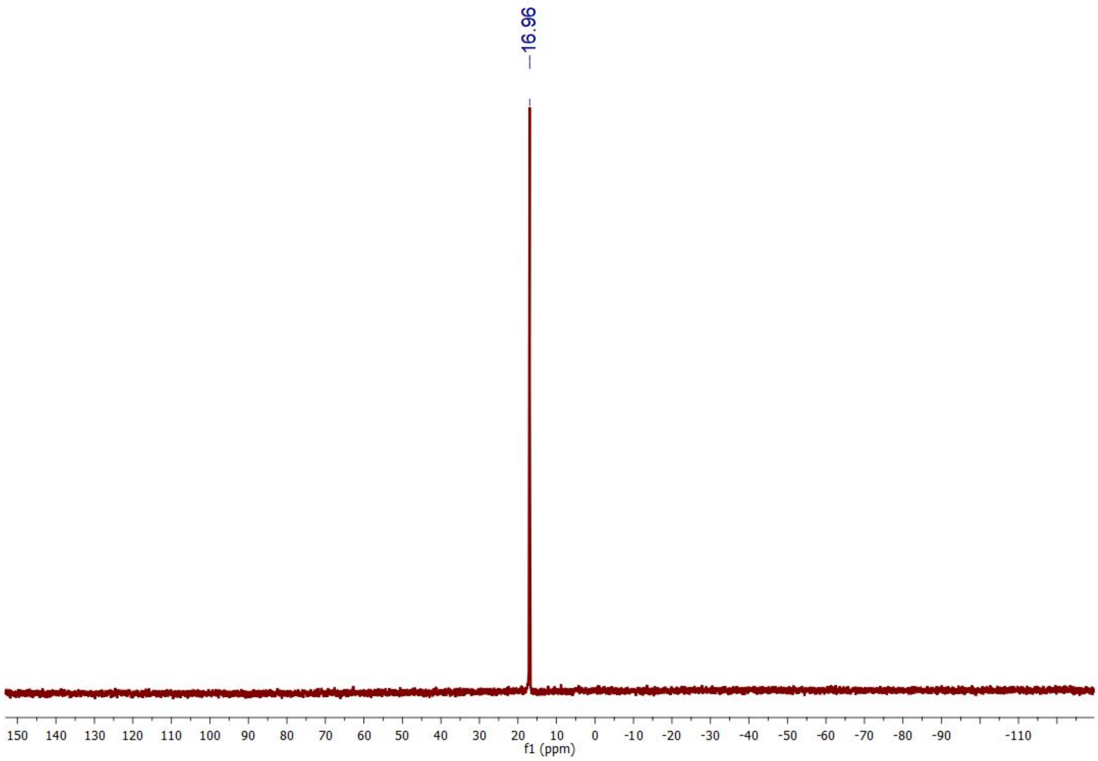

Figure S8. ^{31}P NMR spectrum of the compound **1.3** in $CDCl_3$.

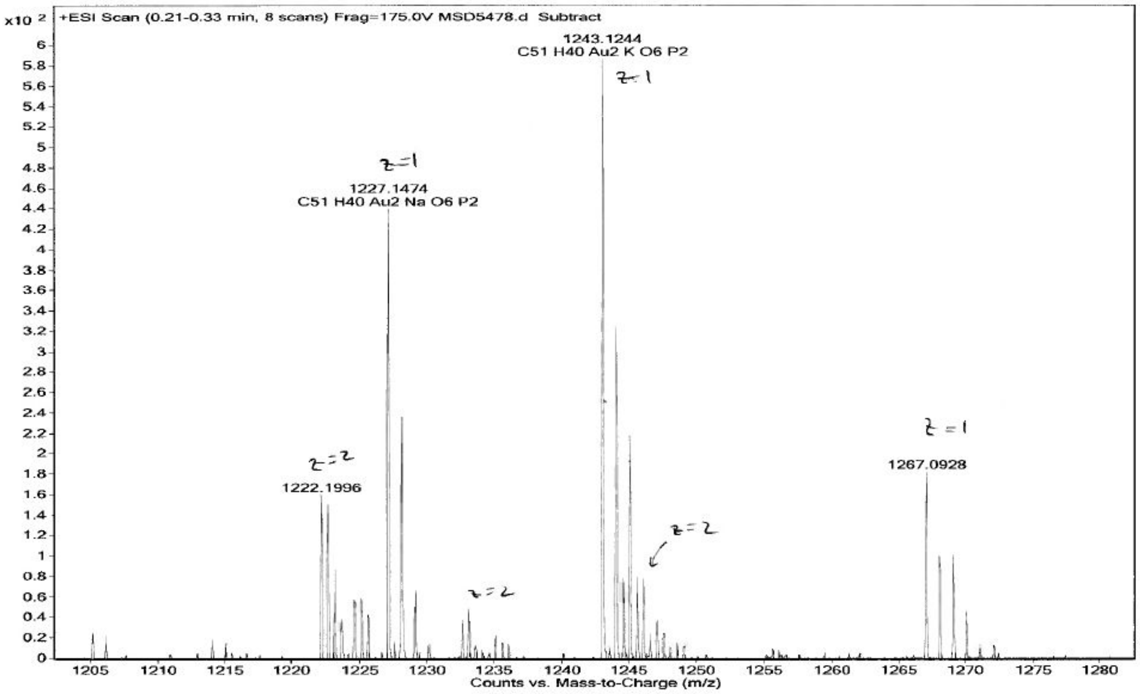

Figure S9. ESI-MS(+) spectrum of the compound **1.1**.

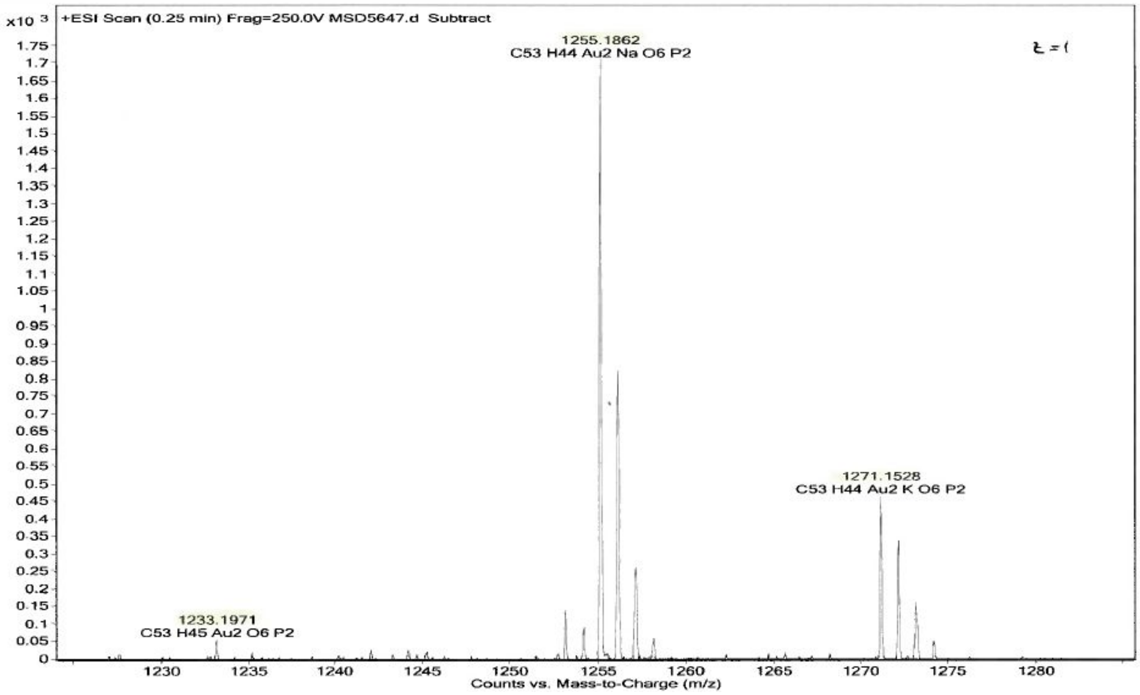

Figure S10. ESI-MS(+) spectrum of the compound **1.2**.

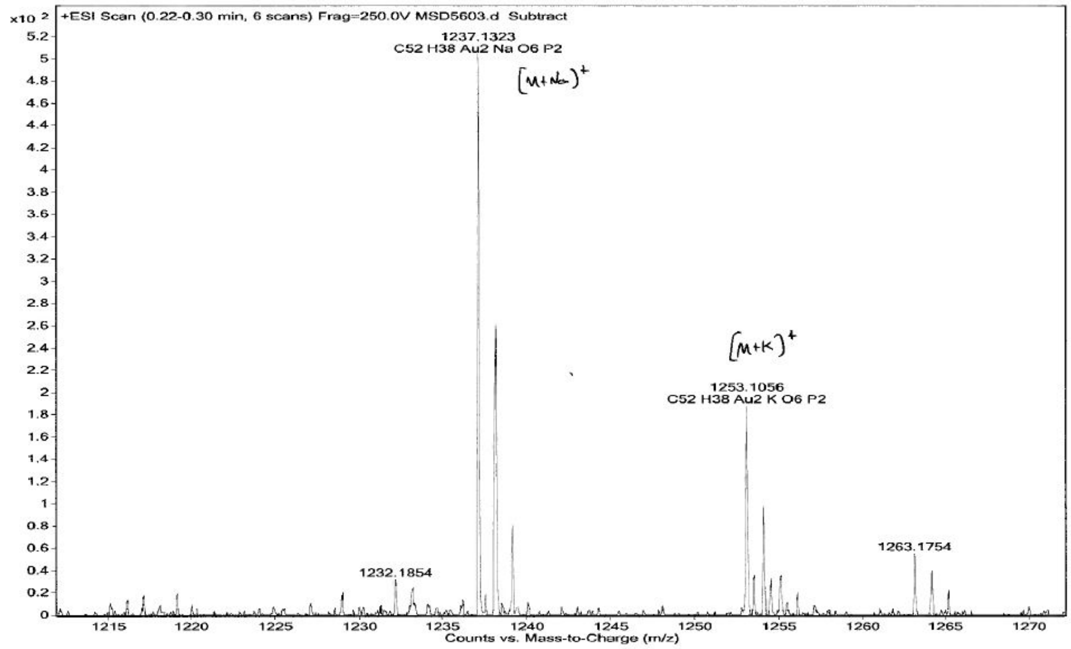

Figure S11. ESI-MS(+) spectrum of the compound **1.3**.

Table S1. Time resolved fluorescence data (lifetimes, τ_i and pre-exponential factors, a_{ij}) obtained with ps-TCSPC technique for gold(I) complexes (**1.1-1.3**) and propynyloxycoumarin ligand (**1**) in different organic solvents; λ_{exc} = 268 nm and λ_{em}=375 nm at T = 293 K.

	Solvent	τ_1 (ns)	a_{i1} (% C$_1$)	τ_2 (ns)	a_{i2} (% C$_2$)	χ^2
	Dx	0.023	1.00	-	-	1.23
	2-MeTHF	0.017	1.00	-	-	1.06
**1**	DMF	0.042	1.00	-	-	1.16
	MeCN	0.029	1.00	-	-	1.12
	DMSO	0.066	1.00	-	-	0.99
	Dx	0.065	0.927 (75)	0.279	0.073 (25)	1.18
	2-MeTHF	0.049	0.954 (70)	0.430	0.046 (30)	1.30
**1.1**	DMF	0.107	0.925 (81)	0.300	0.075 (19)	1.20
	MeCN	0.068	0.917 (76)	0.232	0.083 (24)	1.09
	DMSO	0.137	0.907 (78)	0.379	0.093 (22)	1.12
	Dx	0.061	0.902 (76)	0.174	0.098 (24)	1.04
	2-MeTHF	0.048	0.969 (82)	0.338	0.031 (18)	1.16
**1.2**	DMF	0.102	0.935 (85)	0.253	0.065 (15)	1.11
	MeCN	0.068	0.882 (75)	0.167	0.118 (25)	1.13
	DMSO	0.146	0.912 (80)	0.372	0.088 (20)	0.99
	Dx	0.057	0.957 (84)	0.231	0.043 (16)	1.00
	2-MeTHF	0.042	0.977 (84)	0.348	0.023 (16)	1.33
**1.3**	DMF	0.105	0.965 (90)	0.191	0.035 (10)	1.01
	MeCN	0.066	0.719 (59)	0.116	0.281 (41)	0.84
	DMSO	0.149	0.874 (79)	0.278	0.126 (21)	0.90

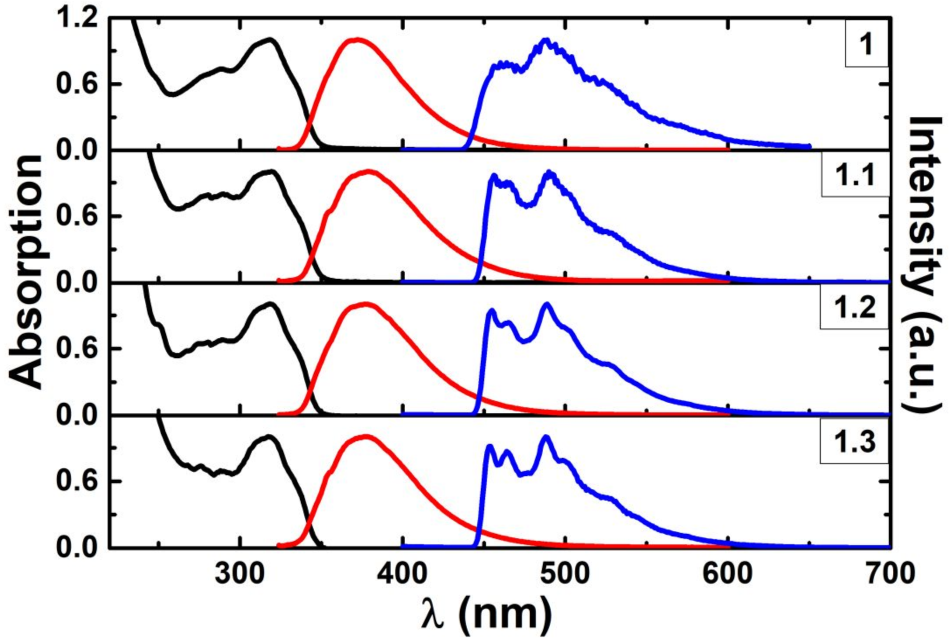

Figure S12. Normalized spectra of gold(I) complexes in 2-MeTHF. Color legend: black line - absorption spectra (293 K); red line - emission spectra at 293 K and blue line - emission spectra at 77 K.

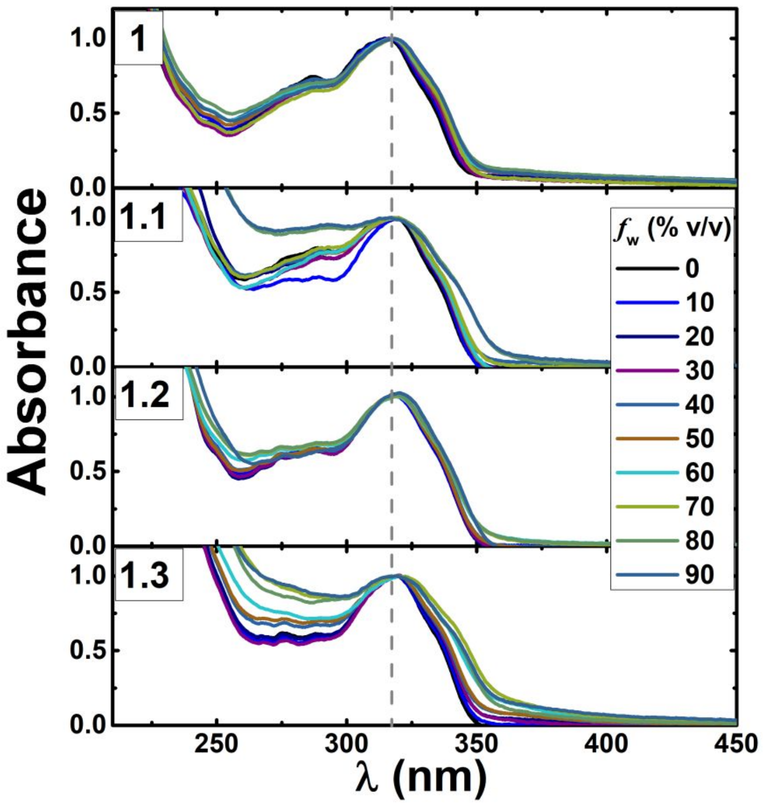

Figure S13. Absorption spectra with increasing water fractions (fw) in MeCN: water mixtures for the three alkynylcoumarin dinuclear gold(I) complexes (**1.1-1.3**) and propynyloxycoumarin ligand, **1**.

Table S2. Time resolved fluorescence data obtained with ps-TCSPC technique for propynyloxycoumarin ligand (**1**) with increasing water fractions (f_w) in MeCN: water; λ_{exc} = 268 nm and λ_{em}=375 nm at T = 293 K.

f_w (% v/v)	τ_i (ns)	χ^2
0	0.084	0.93
10	0.074	1.06
20	0.111	1.01
30	0.138	1.10
40	0.168	0.98
50	0.199	1.09
60	0.293	0.99
70	0.363	1.12
80	0.485	1.13
90	0.585	1.07

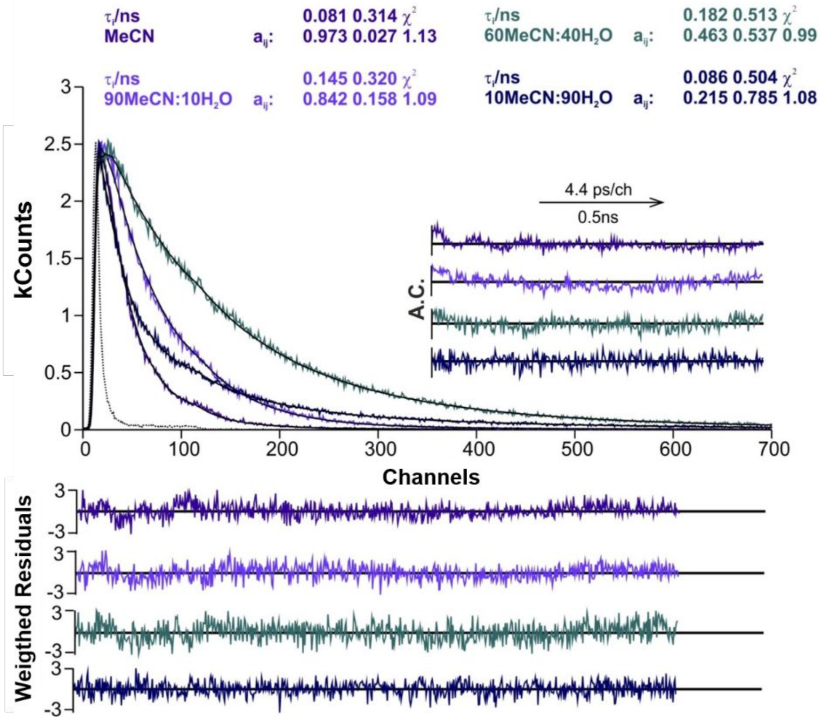

Figure S14. Room-temperature fluorescence decays for **1.1** in different fraction water (in the MeCN: water mixtures) with λ_{exc} = 268 nm and λ_{em} = 375 nm. For a better judgment of the quality of the fit, weighted residuals (W.R.), autocorrelation function (A.C.) and χ^2 values are also presented. The dashed line in the decay of corresponds to the instrumental response function.

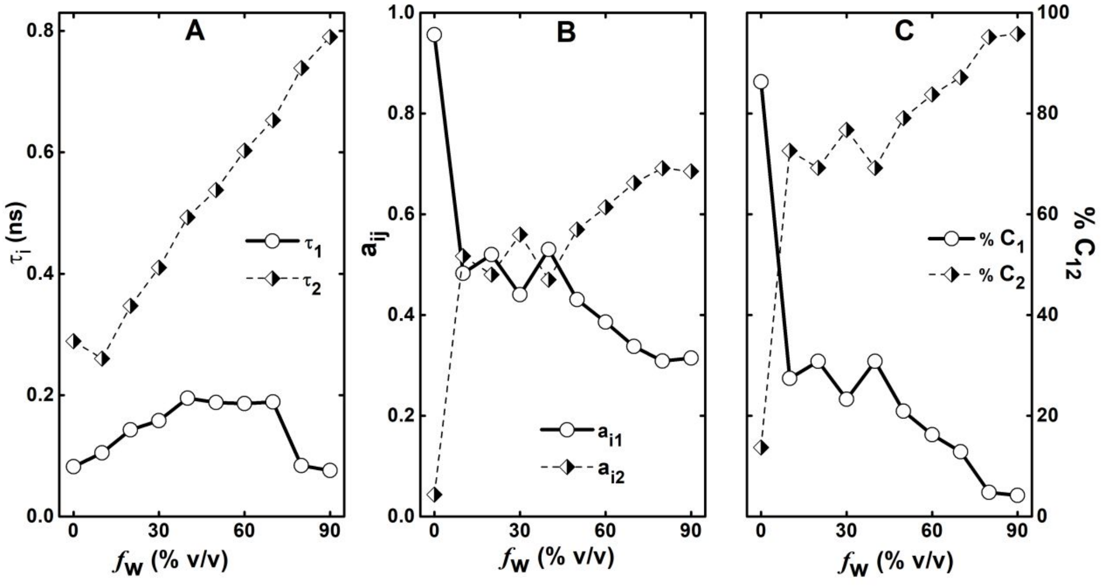

Figure S15. Fluorescence decay parameters obtained for compound **1.1** in MeCN: water mixture with λ_{exc} = 268 nm and λ_{em} = 375 nm. Dependence of (**A**) decay times, (**B**) pre-exponential factors and (**C**) contribution of each species with water percentage (% v/v).

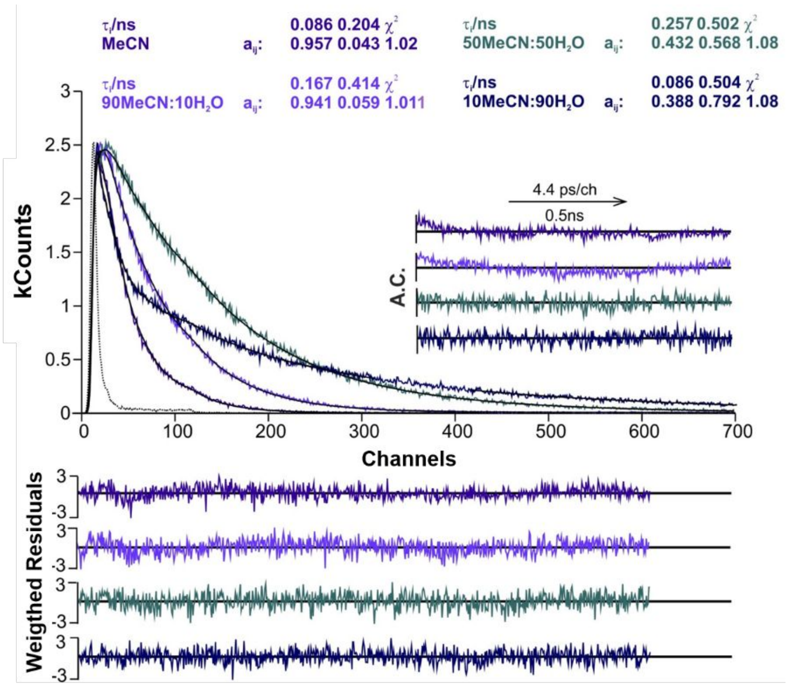

Figure S16. Room-temperature fluorescence decays for **1.2** in different fraction water (in the MeCN: water mixtures) with λ_{exc} = 268 nm and λ_{em} = 375 nm. For a better judgment of the quality of the fit, weighted residuals (W.R.), autocorrelation function (A.C.) and χ^2 values are also presented. The dashed line in the decay of corresponds to the instrumental response function.

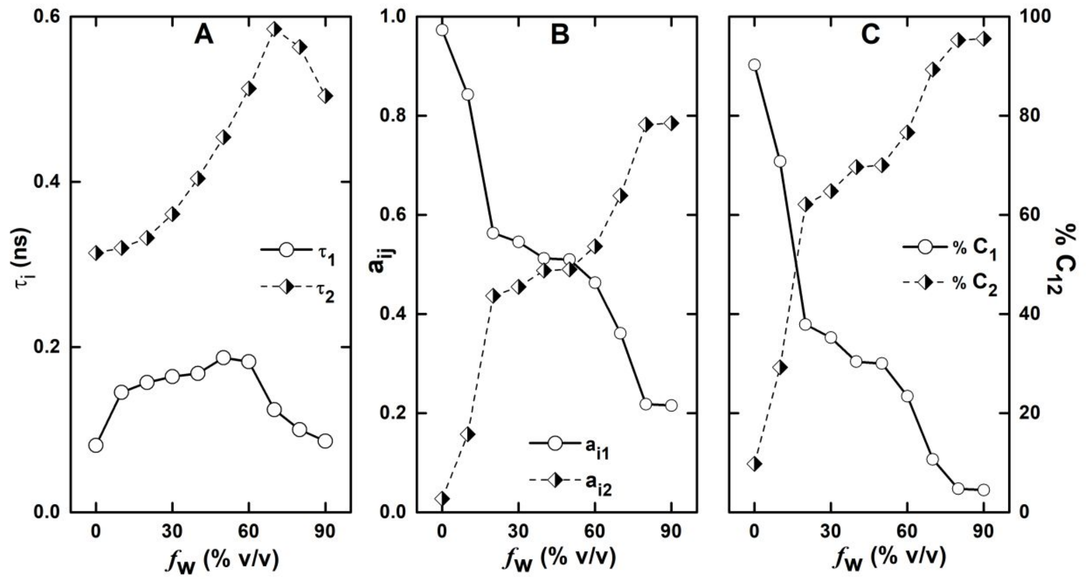

Figure S17. Fluorescence decay parameters obtained for compound **1.2** in MeCN: water mixture with λ_{exc} = 268 nm and λ_{em} = 375 nm. Dependence of (**A**) decay times, (**B**) pre-exponential factors and (**C**) contribution of each species with water percentage (% v/v).

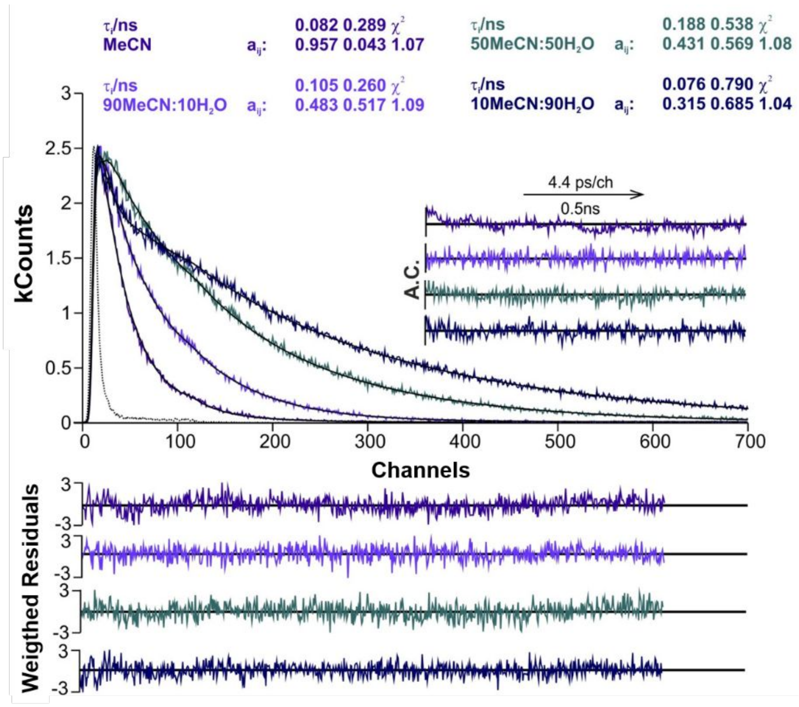

Figure S18. Room-temperature fluorescence decays for **1.3** in different fraction water (in the MeCN: water mixtures) with λ_{exc} = 268 nm and λ_{em} = 375 nm. For a better judgment of the quality of the fit, weighted residuals (W.R.), autocorrelation function (A.C.) and χ^2 values are also presented. The dashed line in the decay of corresponds to the instrumental response function.

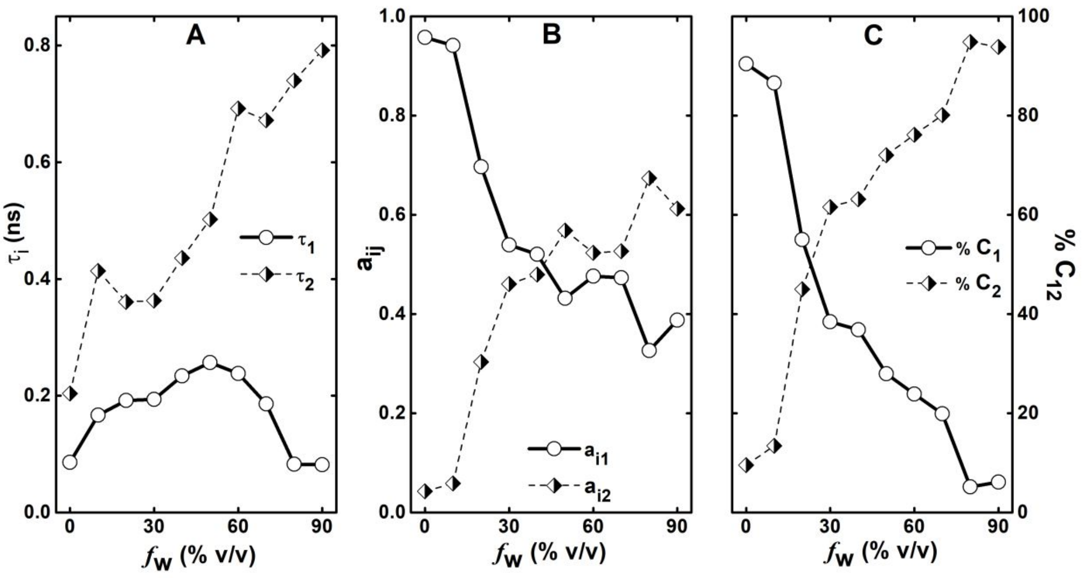

Figure S19. Fluorescence decay parameters obtained for compound **1.3** in MeCN: water mixture with λ_{exc} = 268 nm and λ_{em} = 375 nm. Dependence of (**A**) decay times, (**B**) pre-exponential factors and (**C**) contribution of each species with water percentage (% v/v).

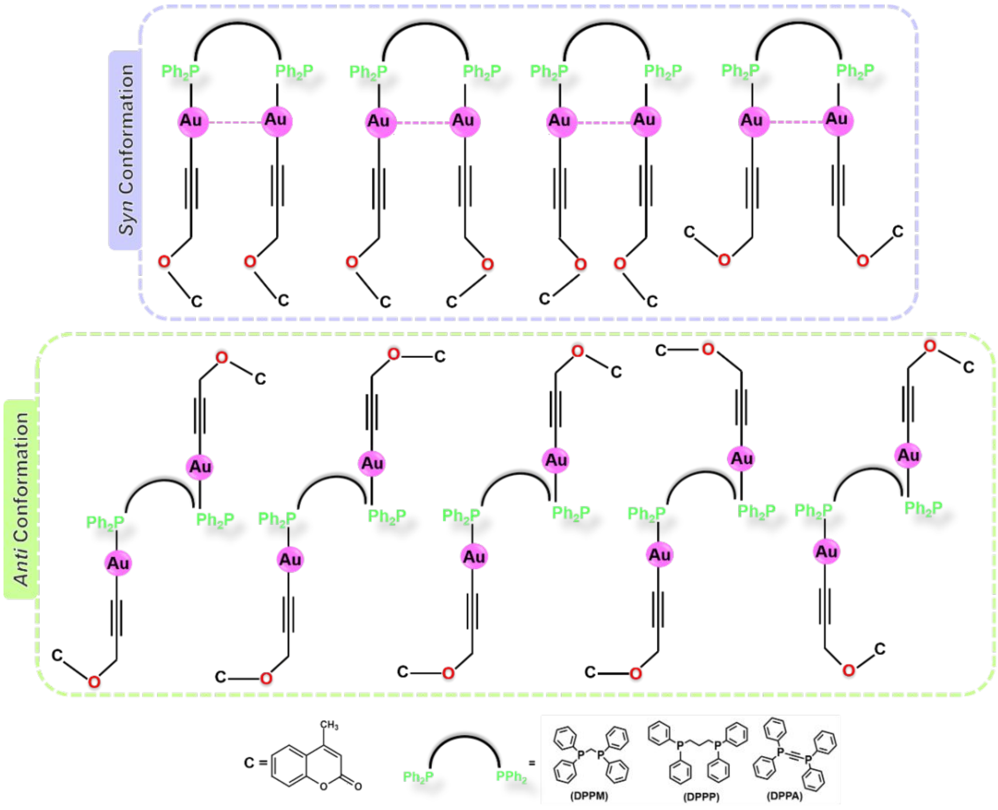

Figure S20. Simplified scheme of the representation of the conformational possibilities with syn vs. anti conformation that each compound can adopt.

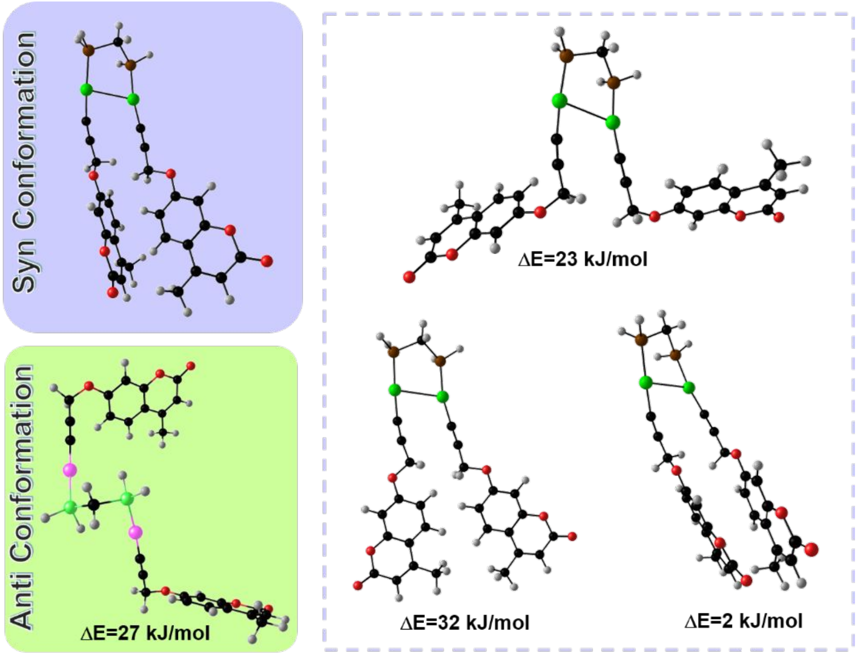

Figure S21. Model compounds to probe the conformational space (with syn and anti conformation) of **1.1**. See text and **Figure S20** for more details.

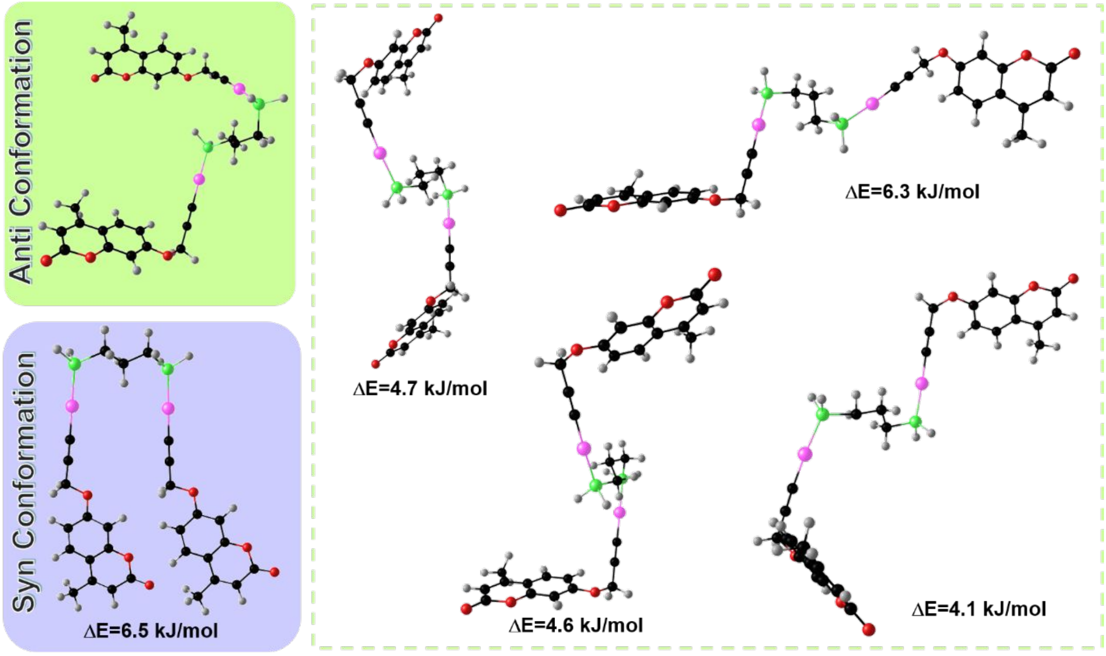

Figure S22. Model compounds to probe the conformational space (with syn and anti conformation) of **1.2**. See text and **Figure S20** for more details.

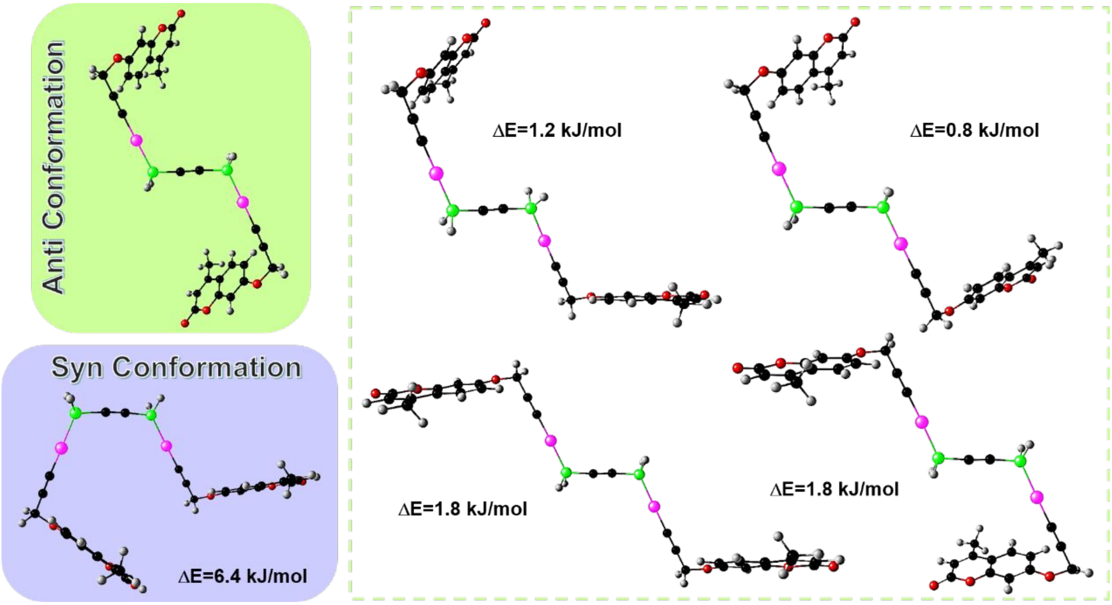

Figure S23. Model compounds to probe the conformational space (with syn- and anti-conformation) of **1.3**. See text and **Figure S20** for more details.

Table S3. Absorption maximum experimental (Exp.) and theoretical (Calc.) values, in different solvents, for organic ligand (**1**) and gold(I) complexes. The oscillator force (*f*) and the respective electronic transition (Trans.) are reported together with the data obtained at the level theory of the DFT//LC-BPBE(ω=0.2)/SBKJC.

	Solvent	Exp.	Calc. (*f*)	Trans.	Exp.	Calc. (*f*)	Trans.
	Dx	317	310 (0.482)	H→L	NO	278 (0.051)	H-1→L
	2-MeTHF	318	311 (0.494)		282	279 (0.037)	
**1**	DMF	316	312 (0.504)	ILCT	NO	280 (0.032)	ILCT
	MeCN	313	311 (0.489)	π→π*	281	280 (0.029)	n→π*
	DMSO	318	311 (0.502)		NO	280 (0.032)	
	Dx	313	314 (1.036)	H-2→L	NO	286 (0.205)	H→L+2
	2-MeTHF	320	313 (1.035)		289	288 (0.174)	
**1.1**	DMF	319	313 (1.046)		NO	286 (0.205)	σ→π*
	MeCN	318	313 (1.020)	MLCT + ILCT	289	286 (0.200)	MLCT
	DMSO	315	313 (1.041)	σ→π* + π→π*	NO	286 (0.212)	
	Dx	320	314 (0.781)	H-1→L	NO	280 (0.046)	H-6→L+1
	2-MeTHF	318	314 (0.862)		289	281 (0.034)	
**1.2**	DMF	320	314 (0.927)	ILCT	NO	281 (0.031)	σ→π*
	MeCN	314	313 (0.870)	π→π*	282	281 (0.028)	MLCT
	DMSO	315	314 (0.921)		NO	281 (0.031)	
	Dx	321	314 (0.777)	H→L+1	NO	281 (0.051)	H-4→L+1
	2-MeTHF	318	314 (0.716)		282	281 (0.037)	
**1.3**	DMF	321	314 (0.730)	ILCT	NO	281 (0.034)	σ→π*
	MeCN	319	313 (0.731)	π→π*	288	281 (0.032)	MLCT
	DMSO	321	314 (0.694)		NO	281 (0.034)	

*NO: not observed due to solvent cut-off

Table S4. Distance Au(I)···Au(I) (in Å), in the different solvents, calculated at the DFT//LC-BPBE (ω=0.2)//SBKJC level for complex **1.1**.

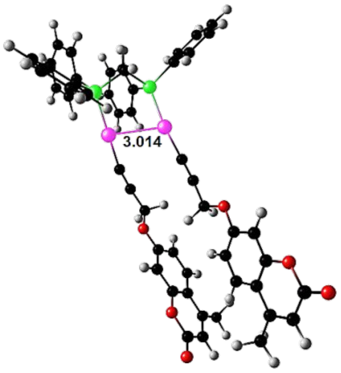

Solvent	Au(I)···Au(I), Å
Dx	2.991
THF	3.010
DMF	3.016
MeCN	3.014
DMSO	3.013

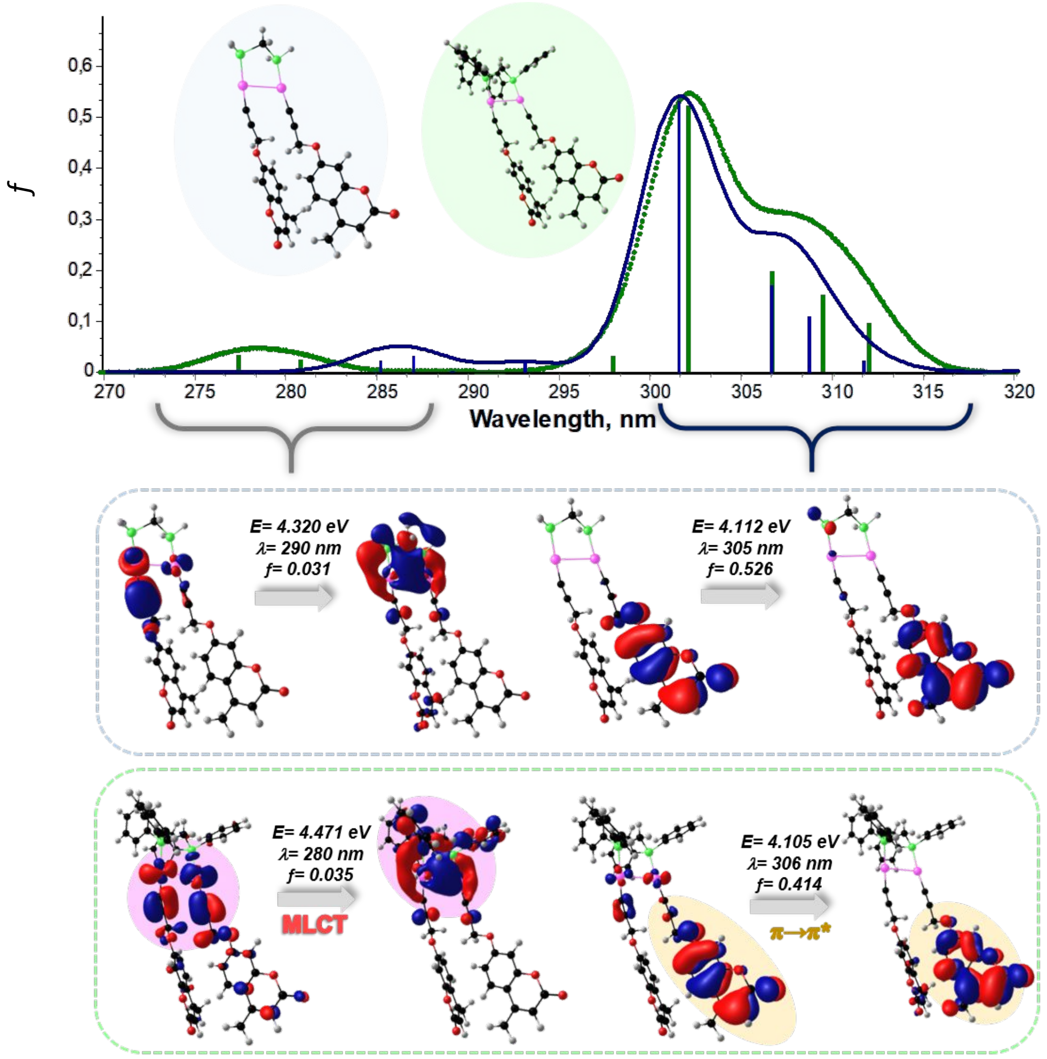

Figure S24. TD-DFT absorption spectra (legend colour: blue line - model compound with phosphane phenyl rings replaced by hydrogens; green line**:** model compound with phosphane phenyl rings) and representative MO contours for the two intraligand (IL) bands of complex **1.1**. Right panel, strong at 306 nm (HOMO→ LUMO+3) and left panel, weak at 280 nm (HOMO-6 → LUMO+3).

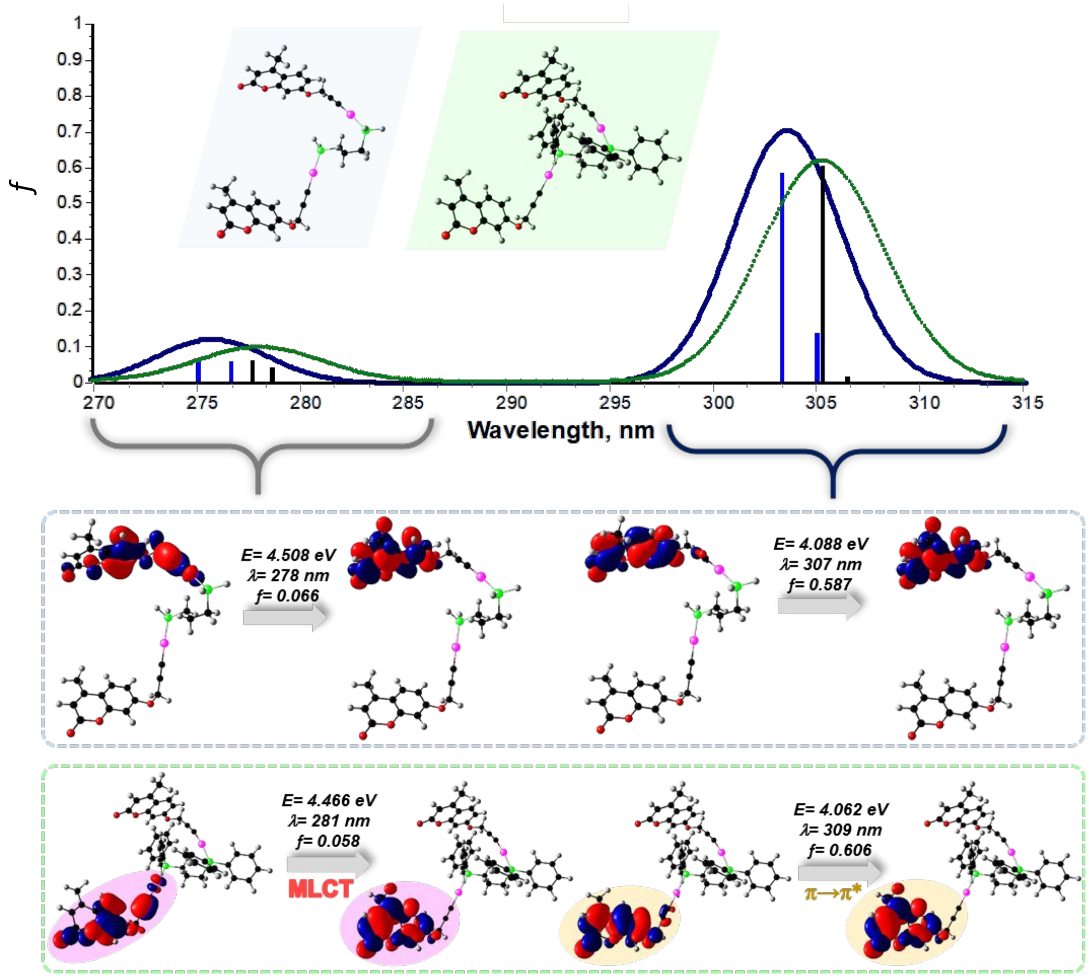

Figure S25. TD-DFT absorption spectra (legend colour: blue line - model compound with phosphane phenyl rings replaced by hydrogens; green line**:** model compound with phosphane phenyl rings) and representative MO contours for the two intraligand (IL) bands of complex **1.2**. Right panel, strong at 309 nm (HOMO→ LUMO+2) and left panel, weak at 281 nm (HOMO-4 → LUMO+2).

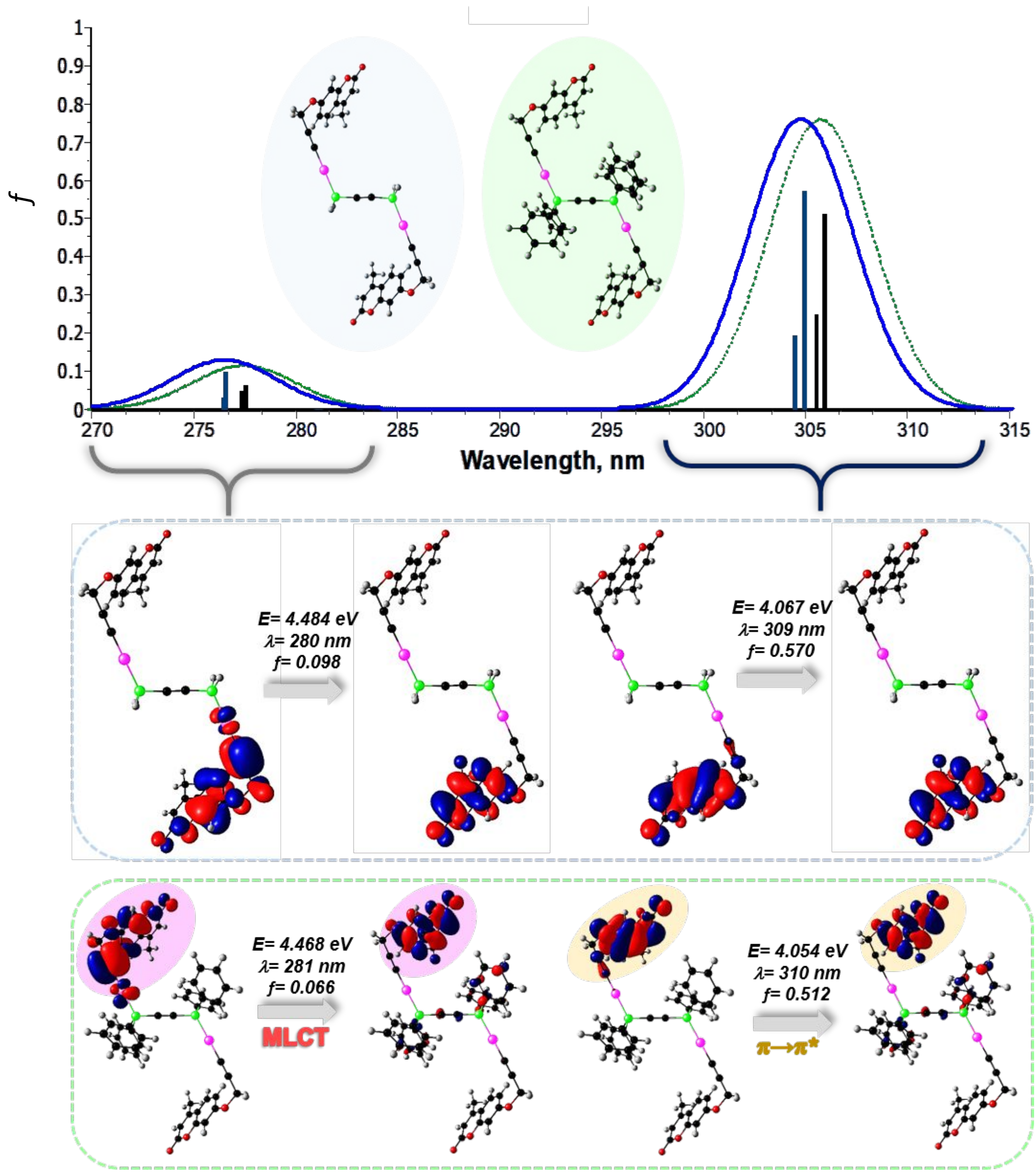

Figure S26. TD-DFT absorption spectra (legend colour: blue line - model compound with phosphane phenyl rings replaced by hydrogens; green line**:** model compound with phosphane phenyl rings) and representative MO contours for the two intraligand (IL) bands of complex **1.3**. Right panel, strong at 310 nm (HOMO-1→ LUMO+2) and left panel, weak at 281 nm (HOMO-5 → LUMO+2).

Table S5. Calculated energies for lowest singlet and triplet states S_1 and T_1 (in eV, and nm in parenthesis) from the ground state S_0 in acetonitrile solution, and phosphorescence emission ($T_1 \rightsquigarrow S_0$), from the relaxed geometry are reported together with the data obtained at the level theory of the DFT//LC-BPBE(ω=0.2)/SBKJC.

cmpd	S_1[a]	S_1*[a]	T_1*[a]
**1**	4.038 (311)	3.4606 (358)	2.7007 (459)
**1.1**	4.016 (313)	3.4410 (360)	2.6837 (462)
**1.2**	4.007 (313)	3.4398 (360)	2.6937 (460)
**1.3**	4.006 (313)	3.4476 (360)	2.6939 (460)

[a]in parentheses, values in nm.

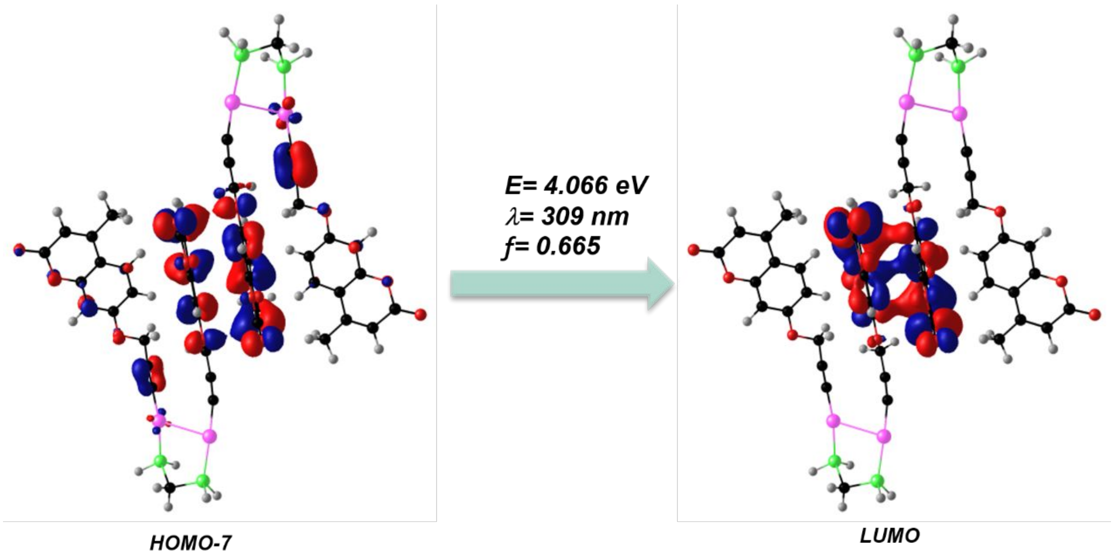

Figure S27. Orbital contours of the HOMO and LUMO for complex **1.1** – "Dimer B" (aggregate).

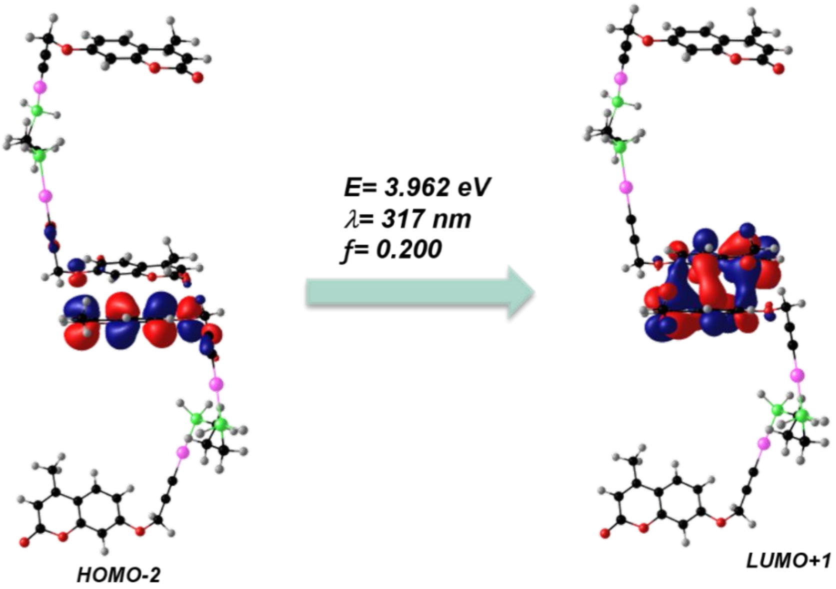

Figure S28. Orbital contours of the HOMO and LUMO for complex **1.2 –** “Dimer B” (aggregate).

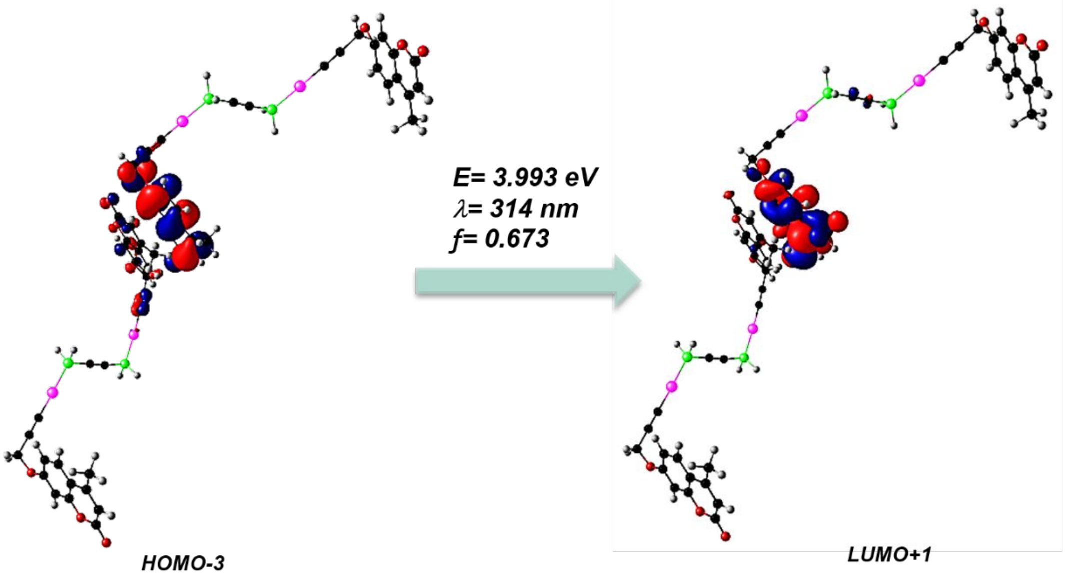

Figure S29. Orbital contours of the HOMO and LUMO for complex **1.3 –** “Dimer B” **(**aggregate).
